# DPCfam: Unsupervised protein family classification by Density Peak Clustering of large sequence datasets

**DOI:** 10.1371/journal.pcbi.1010610

**Published:** 2022-10-19

**Authors:** Elena Tea Russo, Federico Barone, Alex Bateman, Stefano Cozzini, Marco Punta, Alessandro Laio

**Affiliations:** 1 SISSA, Trieste, Italy; 2 AREA SCIENCE PARK, Trieste, Italy; 3 Department of Mathematics and Geosciences, University of Trieste, Trieste, Italy; 4 European Molecular Biology Laboratory, European Bioinformatics Institute (EMBL-EBI), Wellcome Genome Campus, Hinxton, United Kingdom; 5 Center for Omics Sciences, IRCCS San Raffaele Institute, Milan, Italy; 6 Unit of Immunogenetics, Leukemia Genomics and Immunobiology, Division of Immunology, Transplantation and Infectious Disease, IRCCS San Raffaele Scientific Institute, Milan, Italy; 7 ICTP, Trieste, Italy; Fox Chase Cancer Center, UNITED STATES

## Abstract

Proteins that are known only at a sequence level outnumber those with an experimental characterization by orders of magnitude. Classifying protein regions (domains) into homologous families can generate testable functional hypotheses for yet unannotated sequences. Existing domain family resources typically use at least some degree of manual curation: they grow slowly over time and leave a large fraction of the protein sequence space unclassified. We here describe automatic clustering by Density Peak Clustering of UniRef50 v. 2017_07, a protein sequence database including approximately 23M sequences. We performed a radical re-implementation of a pipeline we previously developed in order to allow handling millions of sequences and data volumes of the order of 3 TeraBytes. The modified pipeline, which we call DPCfam, finds ∼ 45,000 protein clusters in UniRef50. Our automatic classification is in close correspondence to the ones of the Pfam and ECOD resources: in particular, about 81% of medium-large Pfam families and 72% of ECOD families can be mapped to clusters generated by DPCfam. In addition, our protocol finds more than 14,000 clusters constituted of protein regions with no Pfam annotation, which are therefore candidates for representing novel protein families. These results are made available to the scientific community through a dedicated repository.

## Author summary

Only a small fraction of proteins for which the sequence is known can be linked to some functional annotation. Identifying evolutionary relationships (homology) between protein regions and grouping them into families can greatly increase the number of proteins for which testable functional hypotheses are available. Indeed, homologous proteins may still share functional features that were originally found in their long-lost common ancestor. While many dedicated protein family databases exist, a large portion of the sequence space remains unclassified. Here, we show that our DPCfam protocol can automatically cluster protein regions from more than 20 million protein sequences in the UniRef50 database and that many of the so-obtained clusters display family-like features. Importantly, our protocol finds more than 14,000 clusters constituted of protein regions with no annotation in the Pfam database, which are therefore candidates for representing novel protein families. These clusters are made available to the scientific community through a dedicated repository and could serve as a go-to list to speed up manual family classification of sequences in the UniProtKB repository. In the future, the DPCfam protocol could be of particular interest for the identification of homologous relationships in large, poorly annotated datasets such as those produced by metagenomics projects.

## 1 Introduction

The number of proteins for which a sequence is known is growing rapidly. The UniProtKB database, for example, contains more than 225M sequences mostly from large-scale sequencing projects but increasingly including high-quality sequences from metagenomic assembled genomes [1]. For their part, dedicated metagenomic databases such as the EBI MGnify [2] contain hundreds of millions of additional sequences although only a fraction of them annotated as full-length.

Knowing the sequence of such a large number of proteins can be of great help in phylogeny reconstruction; however, for most other applications what is of real interest are the functions of the proteins that they represent. Unfortunately, experimental characterization of proteins’ functions remains a painstakingly slow process and, therefore, the gap between protein sequence and functional knowledge is widening.

Thankfully, proteins can be grouped into families according to their evolutionary relationships whereby proteins in the same family have a certain chance of performing similar functions, presumed to descend from their long-lost common ancestor [3]. While it is certainly not always the case that homologous proteins have similar functions, when handled with care, homology is a powerful tool and often represents the first step in the generation of functional hypotheses for yet uncharacterized proteins [4]. For this reason, databases that compile lists of protein families have flourished over the past 25 years. Most of these resources infer homology from sequence similarity although structural knowledge is often utilized to annotate more remote relationships. An important distinction is the one between databases that build families based on full-length sequence similarity and those that instead focus on local similarities. Observing significant similarity between two proteins along their full (or close to full) sequence length allows building a stronger case for their overall functional similarity; however, it also limits considerably the breadth of annotation transfer, typically to more closely related proteins. Targeting local sequence similarities, instead, allows identification of shared evolutionary modules that may represent the only homologous regions between otherwise unrelated proteins, as well as potentially constituting their only functional link. Such evolutionary modules generally map to globular or transmembrane structural domains; however, evolutionarily-conserved long disordered protein modules are also known to exist (in the literature sometimes referred to as unstructured domains) [5].

Existing domain family databases often target specific regions of the protein sequence space focusing, for example, on extracellular or signalling proteins (SMART [6]), enzymes (SFLD [7]), proteins of known structure (several databases including ECOD [8]). Pfam [9] is a database that instead aims to classify all protein types with no strong bias for a given area of the protein universe. Finally, ‘meta-databases’ such as InterPro [10] and CDD [11] integrate information from many other databases with their own annotations. Despite all these remarkable efforts, a large portion of the sequence space is still untouched by family classifications. For example, the Pfam release 35 contained 19,632 families which altogether covered 52.65% of all amino acids in UniProtKB proteins, and left 24% of proteins without annotation on their entire length. Also, annotation levels of metagenomic protein sequence datasets are usually much lower than the ones obtained for UniprotKB and reference proteome sequences. This is due to the fact that metagenomics projects typically target areas of the protein universe that have so far been under-sampled. Indeed, sequences that have been used for family building thus far have been biased toward the culturable set of organisms. There are several other reasons why family classifications are incomplete including the difficulty for sequence profiles built from multiple sequence alignments to capture highly diverse families.

One important obstacle toward a more pervasive family annotation is the fact that all existing resources rely, at least to some degree, on manual supervision. While the latter increases annotation quality, it also inevitably slows down the pace at which novel families are created. A solution to the problem could be represented by an unsupervised, fully automated approach to protein domain family classification. Depending on context, such an approach could be used to rapidly identify the most interesting novel families in large, poorly annotated datasets such as those produced by metagenomics projects or, alternatively, as a go-to list to speed up manual classification of more established datasets such as UniProtKB. Although efforts in this direction have been produced in the past, they have often focused on full-length protein classification (e.g. MCL [12]). Exceptions were ADDA [13] and EVEREST [14]. ADDA was used by Pfam to automatically generate the Pfam-B database until 2015 but subsequently dismissed because of its high computational costs. EVEREST is no longer maintained. We note that recently the Pfam-B database has been reintroduced by Pfam; Pfam-B is now automatically built running the MMseqs2 clustering algorithm [15] on sequences or fragments of sequences lacking Pfam-A annotation [9]. The current Pfam-B building methodology produces only relatively low quality families which require a significant amount of manual curation to be brought up to the level of high quality Pfam-A families.

In what follows, we discuss the application of our new method DPCfam to the automatic classification of protein families in the UniRef50 sequence database. DPCfam is based on local sequence similarity searches followed by clustering using Density Peak Clustering. While we previously published a small-scale experiment that focused on family classification of proteins contained in only two Pfam clans/superfamilies [16], this is the first time that we discuss the use of DPCfam for wholesale classification of a large (about 23 million sequences) sequence dataset. Performing this clustering required developing a dedicated pipeline designed from scratch to ensure memory scaling.

## 2 Materials and methods

### 2.1 Overview of the DPCfam algorithm

DPCfam is a Density Peak Clustering [17] (DPC)-based algorithm that automatically generates protein families starting from all versus all local, pairwise protein sequence alignments. Clustering of local alignments means that DPCfam can, in principle, identify families representing individual evolutionary modules or domains. The same algorithm was already described in [16], where it was applied to two relatively small Pfam clans (4,083 and 2,022 sequences, respectively). In this work, we utilize DPCfam to cluster the entire UniRef50 database (v. 2017_07), containing about 23 million sequences that share no more than 50% sequence identity between each other. Clustering such a large number of sequences required modifying the original DPCfam pipeline in order to increase efficiency of CPU and memory usage.

Starting from local pairwise sequence alignments obtained by BLAST [18], DPCfam performs a two-step DPC clustering followed by a merging procedure aimed at reducing redundancies. The output of the method is a set of *metaclusters*. In [16] and in Results, we provide evidence that sequences belonging to the same metacluster are likely to share a core evolutionary module and thus metaclusters can often be assimilated to protein families. DPCfam output can be used ‘as is’ or, alternatively, metaclusters can be the basis from which to build profile-HMMs for more sensitive searches of the sequence space [19].

**Fig 1:**
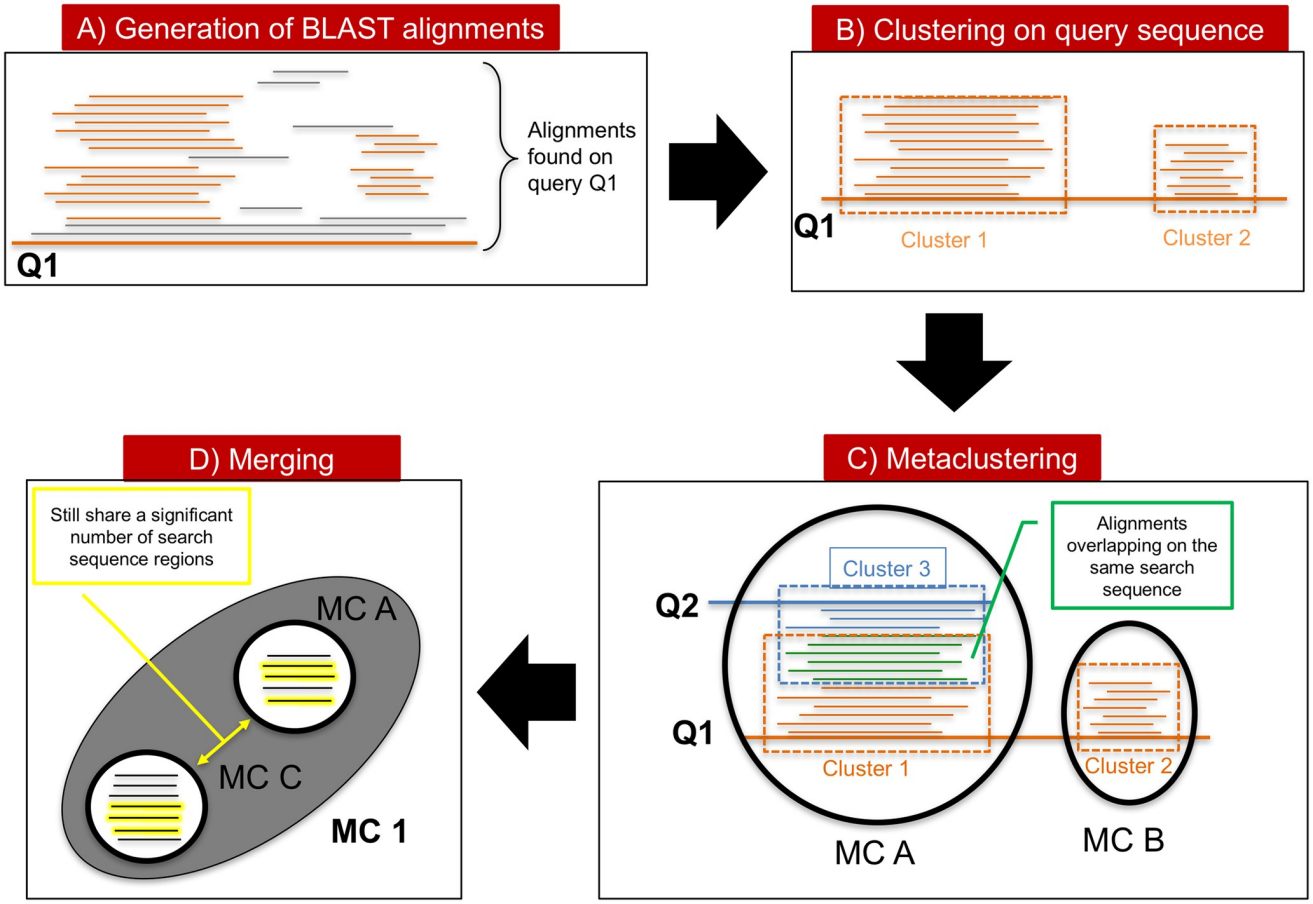
Graphical sketch of the DPCfam workflow. (A) for each query sequence in the reference dataset (UniRef50), we collect all search sequence regions (SSRs) that align to it (BLAST with E-value < 0.1); (B) we use Density Peak Clustering (DPC) to group together SSRs that align to similar portions of the query; (C) primary clusters obtained in (B) for all queries are “metaclustered” (using, again, DPC) according to the number of SSRs they share; (D) finally, metaclusters that still share a significant number of SSRs undergo a further merging step. Material from: ‘Russo et al., Density Peak clustering of protein sequences associated to a Pfam clan reveals clear similarities and interesting differences with respect to manual family annotation, BMC Bioinformatics, published 2021, BioMed Central Ltd.’ [16].

In the following, we recapitulate the DPCfam algorithm (see Fig 1 for a graphical sketch of the method’s workflow) pointing out, when present, differences with respect to our previous implementation.

### 2.2 Generating BLAST alignments

In [16] the first step of the algorithm entailed running BLAST alignments of a relatively small query set (representing a Pfam clan) against a large database of sequences, the “search set”. In this implementation, query set and search set are the same large database, namely UniRef50 v.2017_07. We generate local pairwise alignments using the *blastp* program of BLAST+ (v. 2.2.30) suite. We set the E-value threshold for significance at 0.1 and the max_target_seqs option to 200,000 (that is, we retain no more than 200K significant alignments for each query). We obtain about 55 billion significant local pairwise alignments. Note that the rather liberal threshold on the E-value is meant to favour sensitivity over specificity in the recovery of ‘true’ homologous relationships. In other words, we are willing to accept a small fraction of false positives (out of our 55 billion hits) in order to collect as many true positives as possible. BLAST was run on our local cluster featuring Xeon E5–2680 v2 nodes (2 sockets, 10 cores, 2 threads, 40GB of RAM). We performed about 100,000 blastp batch searches, with each batch containing 200 query sequences. The sequence batches that we use as input to BLAST are obtained from a randomly shuffled version of the UniRef50 database, which allows to balance the sequence composition of the batches and thus the per-batch running time. Also useful for speeding up BLAST is assigning to each sequence an integer ID. These steps are particularly important when having to run millions of searches. We set the blastp multithreading system to 4 threads (-num_threads 4). This step required 750,000 CPU hours.

### 2.3 Primary clustering

Alignments found with BLAST can be represented as query sequence region—search sequence region pairs. In primary clustering, we group together search sequence regions (SSRs) that align to similar portions of the same query. Given a query sequence *q* and the associated set of BLAST-derived SSRs, we define the distance between any two such SSRs *i* and *j* as:

$$\begin{array}{c} d_{i,j}^{q}=1-\frac{|Q_{i}\cap Q_{j}|}{|Q_{i}\cup Q_{j}|} \end{array}$$ (1)

where $Q_{i}$ is the portion of the query covered by SSR *i*. This distance is 0 if *i* and *j* cover the same portion of the query, namely if $Q_{i}=Q_{j}$; while it is 1 if $Q_{i}$ and $Q_{j}$ do not overlap at all.

Then, for each SSR *i* we estimate the local density *ρ*_*i*_ as

$$\begin{array}{c} \rho_{i}=\sum_{j}\chi \left(d_{i,j}^{q}-\mu_{1}\right) \end{array}$$ (2)

where *χ*(*x*) = 1 if *x* < 0 and zero otherwise. Namely, the density of a SSR *i* is given by the number of SSRs that are found at a distance from *i* smaller than *μ*_1_. We add to the distance a small ( ∼ 10^−5^) pseudo-random noise harvested from a uniform distribution to avoid degeneracy, namely cases in which *ρ*_*i*_ = *ρ*_*j*_. We choose *μ*_1_ = 0.2, according to the rule of thumb in [17]. The sensitivity of the results to the value of *μ*_1_ and of the other parameters was benchmarked and discussed in [16].

We then compute the *δ*_*i*_ of the SSRs,

$$\begin{array}{c} \delta_{i}=\underset{j:\rho_{j}>\rho_{i}}{\text{min}}\;d_{i,j}^{q} \end{array}$$ (3)

namely the distance to the closest SSR that has a higher density.

Once *ρ*_*i*_ and *δ*_*i*_ have been determined, we sort all SSRs by decreasing values of the quantity *γ*_*i*_ = *ρ*_*i*_*δ*_*i*_ and select the top 20. Then, we calculate the difference in gamma values between consecutively ranked SSRs Δ_*i*_ = log(*γ*_*i*_/*γ*_*i*+1_). If no such difference is > 0.5, we keep all 20 SSRs as cluster centers (density peaks). If instead there exist one or more SSRs such that Δ_*i*_ > 0.5 we take as cluster centers the lowest ranked SSR *i* that has a Δ_*i*_ > 0.5 and all those ranked above it. The decision of selecting a maximum of 20 density peaks is based on the assumption that the vast majority of proteins do not feature more than 20 families or combinations of families. While this is not always the case (for example for proteins that contain a large number of repeats), we believe that it is adequate for most cases.

SSRs that are not selected as cluster centers are assigned to the closest cluster center, provided that it is closer than *μ*_1_, otherwise they are not assigned to any cluster and not considered anymore in the analysis.

We performed primary clustering using parallel computing with MPI [20]. MPI usage was limited to passing to each process a number of query sequences (and relative alignments) to cluster, sequentially. This step took around 50,000 CPU hours, resulting in about 33 billion alignments clustered into 27 million primary clusters. We used an optimized binary representation for output files, which allowed representing each SSR using only 16 bytes that encode the start and end position of the SSR, the protein it is derived from, and the primary cluster it is found in. The size of the output of primary clustering was about 500GB.

### 2.4 Metaclustering

The second step of our method consists in a further round of DPC performed this time on primary clusters and producing what we call *metaclusters*, or MCs. This operation constitutes both a computational and a memory bottleneck: one has to process 500GB of input data containing alignments grouped into 27 million primary clusters, leading to a distance matrix of (27 ⋅ 10^6^)^2^ elements (sparse). Performing this operation required developing an *ad hoc* strategy, which we describe below.

#### 2.4.1 Generating the distance matrix between primary clusters

We first define the distance between two primary clusters *c* and *c*_0_, associated to two queries *q* and *q*_0_ as:

$$\begin{array}{c} D_{c,c_{0}}=1-\frac{1}{min(N_{c},N_{c_{0}})}\sum_{m\in S_{c},n\in S_{c_{0}}}\delta_{s_{m}s_{n}}\chi \left(d_{m,n}^{s}-\mu_{d}\right) \end{array}$$ (4)

where *N*_*c*_ and $N_{c_{0}}$ are the number of SSRs in primary clusters *c* and *c*_0_, respectively; $S_{c}$ and $S_{c_{0}}$ represent the sets of SSRs clustered in *c* and *c*_0_ respectively, *s*_*i*_ represents a search sequence id and $\delta_{s_{m}s_{n}}=1$ for *s*_*m*_ = *s*_*n*_, 0 otherwise. $d_{i,j}^{s}$ is defined as in Eq 1 replacing $Q_{i}$ and $Q_{j}$ with segments $S_{i}$ and $S_{j}$ (with S a “search” sequence region and Q a “query” sequence region), and *μ*_*d*_ = 0.2, that is, equal to *μ*_1_ in Eq 1.

The distance between two primary clusters is thus defined based on the overlap between the sets of SSRs that constitute each cluster. In particular, $D_{c,c_{0}}=1$ if the two primary clusters have no overlapping SSR and $D_{c,c_{0}}=0$ if each SSR in the cluster with fewer members has substantial boundary overlap with at least one SSR of the bigger cluster. More specifically, the numerator in Eq 4 is the number of SSRs in primary clusters *c* and *c*_0_ that share the same search sequence ID (*δs*_*m*_*s*_*n*_) and are such that $d_{m,n}^{s}<\mu_{d}$; the denominator is the total number of SSRs found in the smaller primary cluster (namely, the one with fewer SSRs).

**Fig 2:**
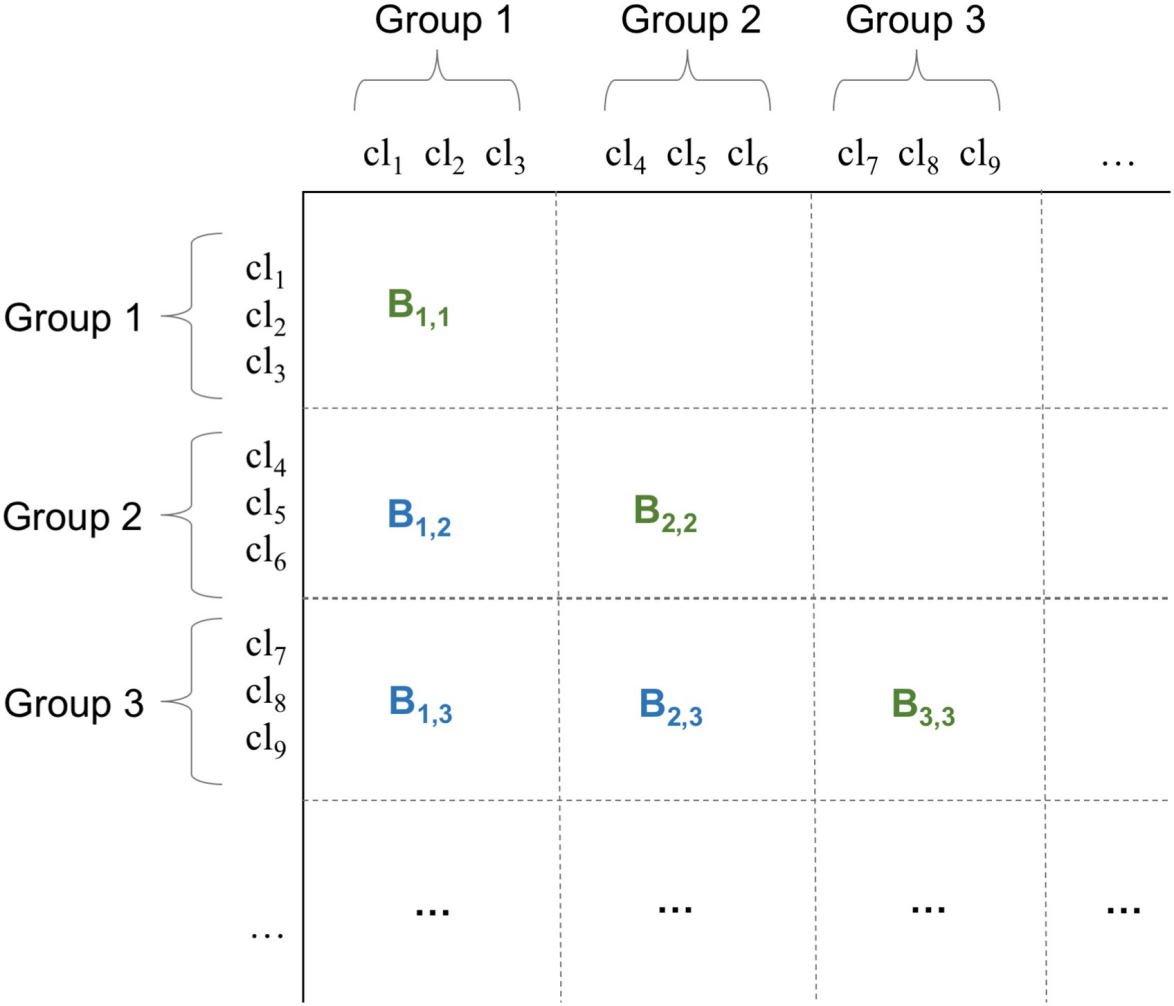
Schematic representation of the primary clusters’ distance matrix organised into blocks. The cluster list is first split into N groups of equal size and then the distance matrix is divided into *N*^2^ blocks with each block containing distances between clusters in two groups. Since the matrix is symmetric, we can consider a single block for each pair of groups. Blue blocks represent off-diagonal blocks, green blocks represent diagonal blocks.

To compute primary cluster distances, we split the full distance matrix *D* into blocks by dividing primary clusters into *N* groups and obtaining *N*^2^ blocks of distances between clusters in each pair of groups (see Fig 2). The content of each block can then be evaluated independently and, the block matrix being symmetric, we have a total of $\frac{N(N+1)}{2}$ blocks to work with. Within each group, we sort the SSRs with respect to the search ID (we use a highly optimized C++ program based on the radix sort algorithm [21]). Such sorting allows placing SSRs with the same search ID, i.e. derived from the same search protein, in contiguous memory sets. Since only pairs of SSRs derived from the same protein can contribute to the distance between primary clusters (see Eq 4), this arrangement allows for a faster computation of all contributions to the distance matrix’s elements. To compute the matrix block *B*_*i*,*j*_, we read simultaneously two files containing the sorted SSRs, one for group *i* and a second for group *j*. As we start reading both files, the first SSRs found in each will have search ID *s*_*i*,1_ and *s*_*j*,1_, respectively. Three cases are then possible:

- *s*_*i*,1_ = *s*_*j*,1_. These two SSRs can contribute to the computation of the distance between the primary clusters they belong to. Since the files are sorted, if there are other SSRs with the same search ID, they will all appear in the following lines. Therefore, we continue reading the files collecting SSRs with this search ID in two separate vectors, *v*_*i*_ and *v*_*j*_ (note: the two vectors may contain a different number of elements). Then, for each SSR in vector *v*_*i*_ we compute the distance $d^{S}$ to any other SSR we collected in *v*_*j*_: if the distance between two SSRs is smaller than 0.2, we add one to the summation in 4.
- *s*_*i*,1_ > *s*_*j*,1_: since SSRs are sorted in both files, in group *i* there is no alignment with a search ID smaller or equal to *s*_*j*,1_. Therefore, we can scroll the lines of group *j* file until we find *s*_*i*,1_ ≤ *s*_*j*,*n*_.
  If *s*_*i*,*n*_ = *s*_*j*,1_, we can compute distances (see previous point).
- *s*_*i*,1_ < *s*_*j*,1_: the same as the previous case, but mirrored.

We continue reading the two files and computing distances between primary clusters following these rules. We can distinguish between off-diagonal blocks and diagonal blocks (see Fig 2). When computing diagonal blocks, we check that we are not working on pairs of SSRs from the same primary cluster, which would be a pointless computation. This allows reducing the number of comparisons to be done within diagonal blocks.

We note that it is very important to reach a balance between the number of groups *N* and their size. Indeed, if *N* is small (*N* = 2 as extreme case), we need to store into memory half of the entire distance matrix; however, on the positive side, we need to compute only three blocks: *B*_1,2_, *B*_1,1_ and *B*_2,2_. With a large *N* we need less RAM for each computation, but we need to compute *N*(*N* + 1)/2 blocks. While having a large number of independent blocks is useful to compute them in parallel, we must consider also that with more blocks we are increasing the I/O burden.

The above described block strategy allows for efficient, parallel computing of the primary clusters’ distance matrix. To further speed up the calculations within each single block, we used a multi-threading strategy. In particular, we used a consumer-producer scheme [22], with *C* consumer threads and *P* producer threads. “Producer” threads scrolled through the input files, computing partial values for distance entries as they found sets of SSRs with same search IDs; “consumer” threads stored these values in a dedicated data structure, representing the block matrix, which is sparse. Reasonable values of *N*, *C* and *P* strongly depend on the features of the supercomputer used for the calculation. We used 10 nodes with 2 Intel(R) Xeon(R) Gold 6126 CPU @ 2.60GHz (24 cores) and 768GB of RAM. We divided the input data into *N* = 50 groups, computing 1,275 blocks, each using *P* = 3 producer threads and *C* = 20 consumer threads. Each block computation occupied about 500 GB in RAM. The complete computation took about 30,000 CPU hours.

#### 2.4.2 DPC applied to primary clusters

Once the distance matrix is computed, we generate metaclusters by DPC. To do that, we calculate for each primary cluster *i* the local density, *ρ*_*i*_, and its minimum distance to primary clusters of higher density, *δ*_*i*_ (see Eq 3). We estimate the local density similarly to what done previously for clustering individual SSRs:

$$\begin{array}{c} \rho_{i}=\sum_{i^{\prime }}\chi \left(D_{i^{\prime },i}-\mu_{2}\right) \end{array}$$ (5)

where *μ*_2_ = 0.9 was again chosen following the rule of thumb in [17].

To compute *ρ*_*i*_ and *δ*_*i*_ we need to use the distance matrix, which is stored in 1,275 files, of 2 GB each. First, we pre-allocate vectors for each quantity, *ρ*_*i*_ and *δ*_*i*_. Both vectors have a size equal to the number of primary clusters, and the indexes correspond to the cluster ids, *c*_*id*_. Such vectors are sparsely populated, making this approach not particularly efficient in terms of memory usage. On the other hand, this strategy allows for a fast access to the vectors’ elements.

We then loop through each file, saving the relevant information in the vectors as we go. Note that, since *δ*_*i*_ is a function of *ρ*_*i*_, we need to calculate these quantities sequentially: thus, we need to loop twice through all distance matrix files.

Once *δ*_*i*_ and *ρ*_*i*_ are calculated, we select density peaks by choosing as peaks all primary clusters that have *δ* = 1 and *ρ* = 1. We found 504,507 peaks, which have been then used to label the rest of the primary clusters based on the metacluster peak closer to them. If the minimum distance to a peak is bigger than *μ*_2_ = 0.9 we leave that primary cluster without classification (singleton). Note that this step requires a third read of the distance matrix files.

### 2.5 Merging and filtering

The clusters found by DPC typically reflect the hierarchical organization of the probability density from which the data are harvested. If a density peak is split in, say, two sub-peaks separated by a high-density saddle point, the approach will detect this structure and return two separate small clusters. Since this level of description is too fine-grained for the large-scale analysis that we want to perform in this work, we merge closely-related peaks by the procedure described below.

We start by computing, for each pair of metaclusters MC’ and MC”, the following quantity:

$$\begin{array}{c} D_{MC^{\prime },MC^{\prime \prime }}=\frac{2}{N_{MC^{\prime }}N_{MC^{\prime \prime }}}\sum_{c^{\prime }\in MC^{\prime },c^{\prime \prime }\in MC^{\prime \prime }}D_{c^{\prime },c^{\prime \prime }} \end{array}$$ (6)

where *N*_*MC*′_ and *N*_*MC*″_ is the number of primary clusters in MC’ and MC”, respectively. *D*_*MC*′,*MC*″_ is the average of the distances between primary clusters contained in the two MCs, where *D*_*c*′,*c*″_ is computed according to Eq 4. We merge all MC pairs for which *D*_*MC*′, *MC*″_ < 0.9.

This final step requires handling a matrix of size 504,507 × 504,507 (all possible MC pairs), where each element of the matrix occupies 8 bytes of memory (this corresponds to roughly 1TB, exploiting the fact that the matrix is symmetric). After merging, we were left with 210,802 metaclusters. Each of these metaclusters is composed of one or more primary clusters, and each primary cluster is associated to a number of SSRs. As a final step, we perform a post-filtering procedure on the SSRs within each MC in order to remove outliers that are likely to represent noise and, separately, to remove duplicate MC members. In particular, given an MC and a search sequence ID, we remove from the MC all SSRs with that search sequence ID that have little or no overlap with the other SSRs. We remove SSR *i* if no other region *j* exists such that $\delta_{s_{i}s_{j}}\chi \left(d_{i,j}^{S}-\mu_{d}\right)=1$ (cfr. Eq 5). Further, given an MC and a set of overlapping SSRs belonging to the same search sequence, we calculate the average boundaries of the overlapping regions (average start and average end, respectively) and keep as a sole representative of this set the one that best overlaps with such boundaries. The so-obtained set of SSRs is what we call the MC seed. We note that the post-filtering procedures have very little impact on the total computing time of DPCfam.

### 2.6 Expanding MC membership using profile-HMMs

For each MC, we take all SSRs and reduce redundancy at 60 percent sequence identity (Cd-Hit [23]). If more than 5,000 sequences are left after redundancy reduction, we pick 5,000 representative sequences at random. We use the redundancy-reduced set of sequences to build a multiple sequence alignment (MUSCLE V.3 [24], with maxhours parameter set to 1.0 to accelerate the procedure), from which we subsequently derive a profile-HMM (hmmbuild of the HMMER package v. 3.1b2 [19]). Finally, we run the profile-HMM against the UniRef50 database (HMMER-hmmsearch) using standard E-value thresholds (domain E-value = 0.03, protein E-value = 0.01). We take all significant profile-HMM hits as our new MC member regions. On average, we see a 4-fold increase in the number of MC members with respect to the original MC definition, although gains vary considerably from MC to MC. We note that, for 26 MCs, HMMER is not able to build a profile-HMM most probably due to the low quality of their alignments.

### 2.7 Parent-child relationships between MCs

Some MCs are such that several of their members have large overlaps to the members of a second MC. This can happen, for example, if one MC includes two domains and the other only one. For these cases, we introduce the notion of ‘parent-child’ relationship. In particular, given two MCs, we consider all member pairs that are found on the same protein (we use the profile-HMM-based definition of MC members, see Methods’ section *Expanding MC membership using profile-HMMs*). We say that the shorter member of the pair is covered by the longer member if the length of their intersection divided by the length of the shorter member is larger than 0.8. We then calculate the fraction of members, *f*_*mem*_(*i*, *j*), of one MC (i) that cover the members of the other (j) and consider the two MCs in a parent-child relationship if *f*_*mem*_(*i*, *j*) > 0.1 (where i is the child and j is the parent).

### 2.8 Assessing properties of MCs’ search sequence regions (SSRs)

We predict amino acids that are found in Low Complexity [25], Coiled-Coil [26], Disordered [27] or Transmembrane [28] regions using the programs segmasker [29], DeepCoil [30], IUPred2A [31] and Phobius [32], respectively. All programs are run with default parameters. When running IUPred2A, amino acids are considered as disordered if their score is > 0.5. We label MCs that have more than 10% of amino acids from all of their SSRs in a low complexity, coiled-coil or disordered region as Low Complexity MCs, Coiled-Coil MCs and Disordered MCs, respectively. MCs that have more than two predicted transmembrane regions (calculated as an average over all SSRs) are labeled Transmembrane MCs.

### 2.9 Annotation of DPCfam MCs using the Pfam family database

Given a MC, we assign a Pfam ground truth architecture (GTA) to each of its seed SSRs.

In order to do that, we use Pfam-A v.33 [9] annotations of all proteins in UniRef50 v.2017_07 that are also found in UniProtKB v.2019_08 (18,891,393 sequences total, constituting ∼80% of UniRef50 v.2017_07). UniRef50 proteins not in UniProtKB are not considered for this comparison.

The GTA of a sequence $S_{i}$ is the ordered set of Pfam families, if any, that map to $S_{i}$ (note that a one-amino acid overlap is enough to include a Pfam family into the GTA). After GTA assignments, we define the dominant architecture of an MC as the GTA that is most abundant among its seed sequences. The dominant architecture can be calculated using Pfam annotations either at the family (DA, hereafter) or at the clan level (DAC), depending on what the ultimate scope of the mapping is.

DA and DAC can, for example, be used for calculating measures (first defined in Russo et al. [16]) that provide useful insights into homologous relationships between MC seed sequences. %DA (%DAC) is the percentage of seed sequences with a GTA that matches exactly the DA (DAC). In the following, we refer seed sequences matching the DA as “DA seed sequences”. Based on %DA, we separate the MCs into three categories: ‘unknown’ MCs are those with %DA = 0 (that is, none of their seed sequences has a Pfam family annotation); ‘partly-known’ MCs are such that 0 <%DA< 50 and, finally, ‘well-known’ are those MCs that have %DA≥50. Further, we define %DACF (DAC Fewer) of an MC as the percentage of seed sequences that have a GTA featuring no Pfam annotation outside of the clans represented by the DA families (as limit cases, these include sequences that have Pfam annotation matching exactly the DAC and, at the other end of the spectrum, sequences lacking any Pfam annotation). Finally, we add to the seed sequences that fall into %DACF those that have GTAs with one or more families not in the DA clans but that feature at least one family belonging to one of the DA clans and obtain %DACFA (DAC fewer and additional). Note that DAC ⊆ DACF ⊆ DACFA. For each MC, these measures represent the fraction of seed sequences featuring Pfam annotations that either match the DA (%DA and %DAC) or are at least not incompatible with it (%DACF and %DACFA). As such, they can provide important insights into homology relationships within MC members. For example, MCs with %DA or %DAC close to 100 have the vast majority of their seed sequences carrying the same Pfam annotation (at the family or clan level) and thus according to Pfam share to the very least a core homologous region. Even when %DA/DAC values are low and provided that %DACF or %DACFA are high, most members of an MC are still likely to share a core homologous region although this may not match the full DA annotation. Finally, by definition, MCs with low %DACFA values feature a sizable fraction of members that have Pfam annotations completely unrelated to their DA and are thus the ones most likely to contain a mixture of non-homologous regions. In some instances, however, these apparent inconsistencies may instead point to incomplete Pfam annotations, whereby families thus far considered unrelated may have a common evolutionary origin and thus belong to the same Pfam clan. Examples of MCs falling in each of the above categories can be found in Russo et al. [16].

Further, given an MC and its DA, we look at how the MC seed sequence boundaries compare to those of the Pfam DA annotation. For this analysis, we restrict ourselves to MCs with %DA≥ 50 and, within those, to DA seed sequences (see definitions above). For all DA seed sequences $S_{i}$, we call $P_{i}$ the region of the full-length protein they belong to that is covered by their GTA (which, in this case, is the same as the MC’s DA). $P_{i}$ stretches from the first amino acid of the most N-terminal Pfam family in the GTA to the last amino acid of the most C-terminal Pfam family in the GTA. We estimate the overlap between an MC DA seed sequence $S_{i}$ and the GTA/DA $P_{i}$ as follows:

$$\begin{array}{c} O_{S_{i},P_{i}}=\frac{|S_{i}\cap P_{i}|}{|S_{i}\cup P_{i}|} \end{array}$$ (7)

where |.| represents the length (expressed in number of amino acids) of the indicated region. $O_{S_{i},P_{i}}$ ranges between 0 (no overlap) and $min(|S_{i}\left|,\right|P_{i}\left|\right)/max(|S_{i}\left|,\right|P_{i}\left|\right)$ (1 if $S_{i}=P_{i}$). Next, we calculate the average of $O_{S_{i},P_{i}}$ over all the DA seed sequences (*O*_*MC*_). This is our measure for the ‘overlap’, or boundary agreement, between an MC and its DA. Since *O*_*MC*_ is symmetric with respect to MCs and DAs, in order to further investigate the nature of the overlap, for each protein sequence featuring an MC seed sequence-DA pair we introduce two additional quantities:

$$\begin{array}{c} F_{red,i}=\frac{|P_{i}\left|-\right|S_{i}\cap P_{i}|}{|P_{i}|}=\frac{|P_{i}\left|-\right|I_{i}|}{|P_{i}|} \end{array}$$ (8)

$$\begin{array}{c} F_{ext,i}=\frac{|S_{i}\left|-\right|S_{i}\cap P_{i}|}{|S_{i}|}=\frac{|S_{i}\left|-\right|I_{i}|}{|S_{i}|} \end{array}$$ (9)

*F*_*red*,*i*_ represents the fraction of the GTA/DA $P_{i}$ that is not covered by the MC seed sequence $S_{i}$; *F*_*ext*,*i*_ is the fraction of $S_{i}$ not covered by the GTA/DA.

We then average both quantities over all seed sequence-DA pairs obtaining $F_{red}^{MC}$ and $F_{ext}^{MC}$. Following this, we classify MCs into the following 4 categories (see S1(B) Fig):

- *equivalent* : both $F_{ext}^{MC}$ and $F_{red}^{MC}<0.2$, yellow;
- *reduced* : $F_{ext}^{MC}<0.2$ and $F_{red}^{MC}\geq 0.2$, blue;
- *extended* : $F_{ext}^{MC}\geq 0.2$ and $F_{red}^{MC}<0.2$, pink;
- *shifted* : both $F_{ext}^{MC}$ and $F_{red}^{MC}$ are ≥ 0.2, green.

### 2.10 Definition of representative MCs for Pfam and ECOD families

In this section, we describe how we assign to each Pfam or ECOD family a best matching (representative) MC. For finding representative MCs for Pfam families, we first run the MCs’ profile-HMMs against the UniRef50 database (HMMER-hmmsearch) using standard E-value thresholds (domain E-value = 0.03, sequence E-value = 0.01). Then, given a Pfam family, we define its representative MC as the one for which the set of its profile-HMM matches cover at least half of the Pfam family sequence IDs that are found in UniRef50 and, if more than one MC satisfies this condition, the one that produces the highest average overlap (see Eq 7) with the Pfam family. We classify each representative MC as equivalent, reduced, extended or shifted (see above and S1 Fig).

Next, we download ECOD [8] F-group (families with close evolutionary relationship) annotations of PDB proteins from version 20210511 and remove annotations of non-contiguous domains (that is, domains that are split in multiple segments along the protein sequence). We further restrict our analysis to PDB proteins that feature in UniProtKB (referred to as ‘PDB-UniProtKB sequences’, hereafter). We obtain a dataset of 67,786 distinct annotations on 45,518 proteins from a total of 11,733 ECOD families. We then run DPCfam profile-HMMs against the ECOD-annotated PDB-UniProtKB sequences (hmmsearch with domain E-value = 0.03, sequence E-value = 0.01). We finally define the representative MC of each ECOD family (if any) exactly as we did previously for Pfam families.

### 2.11 Assigning AlphaFold DB representative structures to MCs

AlphaFold DB [33, 34] provides protein structure predictions for the human proteome and a host of other proteins. This data represent an opportunity to further characterize MCs at a structural level when no experimental PDB structure for its member regions is available. We map AlphaFold structures to MCs by running the MCs’ profile-HMMs with hmmsearch against the dataset of 992,316 proteins that constitute version 2 of AlphaFold DB. The representative of an MC is chosen as the protein in AlphaFold DB with a significant match to the MC’s profile-HMM (domain E-value = 0.03, sequence E-value = 0.01) and (if more than one match is present) as the one with the highest coverage of the profile-HMM model. If there is no AlphaFold protein satisfying these criteria, no representative is assigned.

## 3 Results

### 3.1 Automatic clustering of protein regions from the UniRef50 database

**Fig 3:**
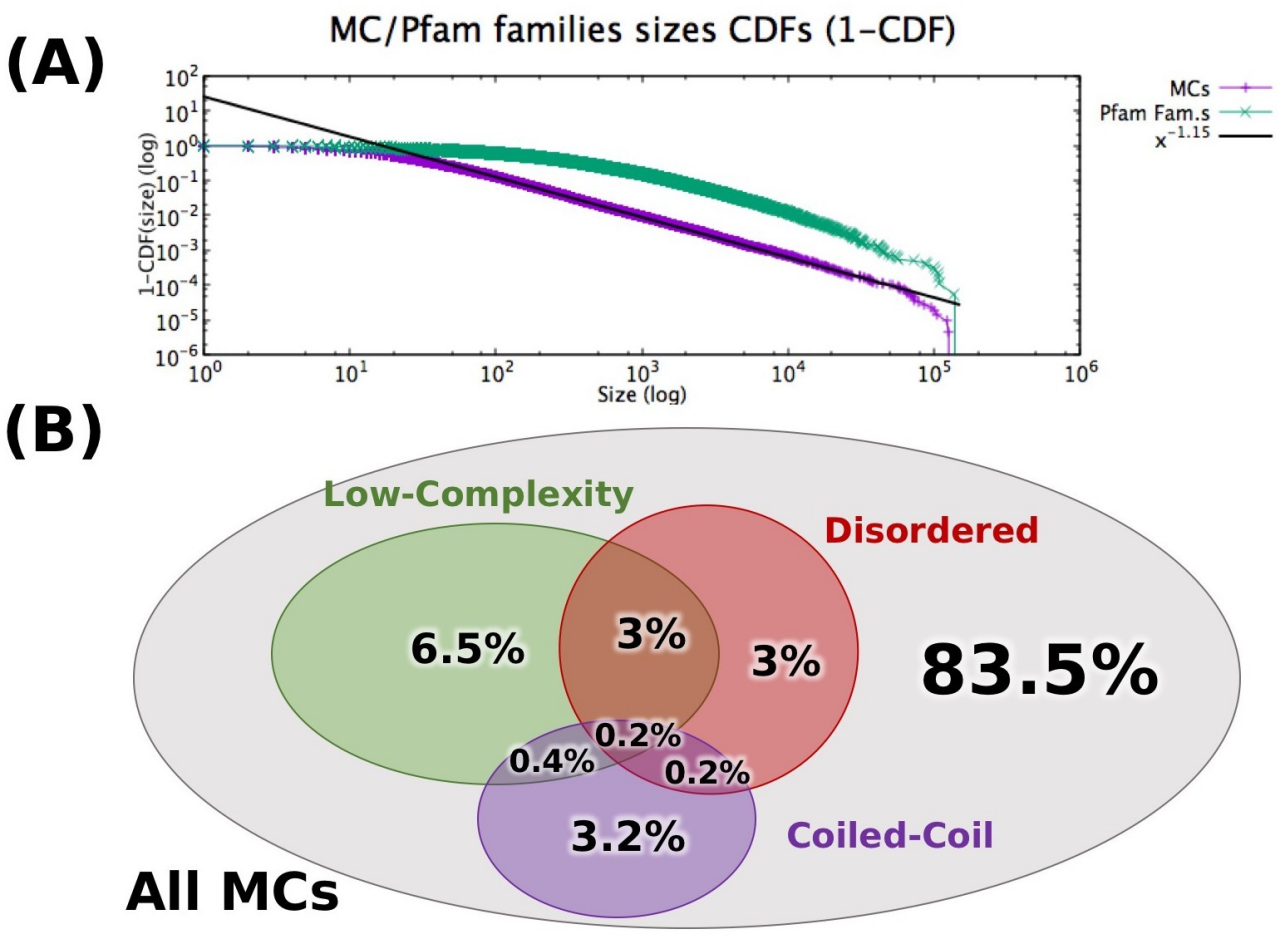
General properties of Metaclusters. **A** Cumulative Distribution Function (1-CDF) of MC (purple) and Pfam family (green) sizes. All 210,802 MCs have been considered, with the size of a MC equal the number of its seed sequences. Pfam families (v. 33) are all those in Uniref50 ∩ UniprotKB (18,189 total) and family size is the ‘full’ size, that is, not only considering Pfam seed sequences. Black line: best fit of MC’s CDF with a power-law (CDF exponent *γ* = −1.15, corresponding to an exponent of −2.15 of the Probability Distribution Function). In S3 Fig, we additionally plot the size of MCs/Pfam families as a function of their size rank. **B**: Venn diagram showing the percentage of Low Complexity MCs (green), Disordered MCs (red) and Coiled-Coil MCs (purple), respectively (see Methods for definitions). Note that, in this case, only MCs containing at least 50 seed sequences and with average length ≥ 50 are considered (46,828 total).

Running DPCfam on the Uniref50 database produced 210,802 Metaclusters (MCs). In Fig 3A (purple), we show that their size is approximately distributed as a power-law with exponent ∼ −2.15 for almost 4 orders of magnitude (black line). The power-law functional form of the distribution of protein family sizes has been ascribed to the biological processes at the basis of protein evolution [35] such as gene duplication or gene generation. A qualitatively similar trend is observed for the size of Pfam families (green) as it has previously been reported [35]. In the case of Pfam families, however, a power-law behaviour is observed for only about 2 orders of magnitude, suggesting potential under-representation of smaller size families in Pfam. Indeed, we note that about 75% of MCs and about 30% of Pfam families have less than 50 seed sequences, respectively. For the rest of our analysis, we will focus on MCs of size ≥50 since they are more interesting in terms of their taxonomic range and because, for this group, comparison with the Pfam classification appears to be more meaningful. We additionally exclude from downstream analysis MCs such that their average seed sequence length is smaller than 50 amino acids. This leaves us with 46,828 MCs, or 22.2% of the total. We note that most of these MCs feature a rather uniform distribution of seed sequence lengths. Indeed, the ratio between standard deviation and average of the length of their seed sequences is typically around 0.1 and only in 5.9% of the cases it is larger than 0.2. Also, the median of the MCs’ average seed sequence length is 99.9, with only about 4% of MCs having average length >500.

Out of a total of 46,828 MCs, 16.5% are characterized by high content of one or more among low-complexity, disordered and coiled-coil regions (Fig 3B). All of these regions present biases in their amino acid composition that make it harder, at least in principle, to infer homology from sequence similarity; additionally, disordered regions typically present a higher than average number of insertions and deletions, which further complicate the task of aligning them properly. Still, high disorder levels do not necessarily affect the quality of a MC. The existence of conserved protein families rich in disordered regions is well known from the literature [5] and, indeed, some disordered MCs show high levels of sequence conservation (see S2 Fig for an interesting example of a disordered MC). At any rate, 83.5% of MCs have low contents of compositionally-biased regions, suggesting that they may correspond to the structured portion of the protein universe and thus represent prime targets for family building. 3.5% of MCs are predicted to contain at least two transmembrane helices and have low contents of compositional bias thus likely spanning transmembrane helical bundle domains.

### 3.2 Homologous relationships between member regions of DPCfam MCs

In order to investigate homology relationships between MC members, we look at their Pfam family annotation. To each MC, when possible, we assign a Pfam dominant architecture (DA; note that it may correspond either to a single family or to a multi-family architecture). For 14,190 MCs we find no Pfam annotation in any of their seed sequence regions and as a consequence we cannot assign a DA to them. We label these MCs as ‘unknown’. We will discuss in one of the following sections how unknown MCs may represent a potential pool of novel families. For all the others, we calculate the proportion of seed sequences with Pfam annotations matching the DA exactly (%DA) or partially (%DAC, %DACF and %DACFA) (Methods). Overall, 11,429 MCs have %DA> 95 (this is 35.1% of those with a DA). These are MCs that can be considered as almost completely covered by existing Pfam families. An additional 62.6% of our MCs with a DA are such that %DAC< 95 and %DACFA> 95. As explained in Methods, member regions of these MCs are very likely to share a core homologous region. In conclusion, only about 2.3% of MCs to which a DA can be assigned have %DACFA< 95 and are thus, according to Pfam, a potential mixture of non-homologous regions.

### 3.3 Assessing the quality of MC boundaries

**Fig 4:**
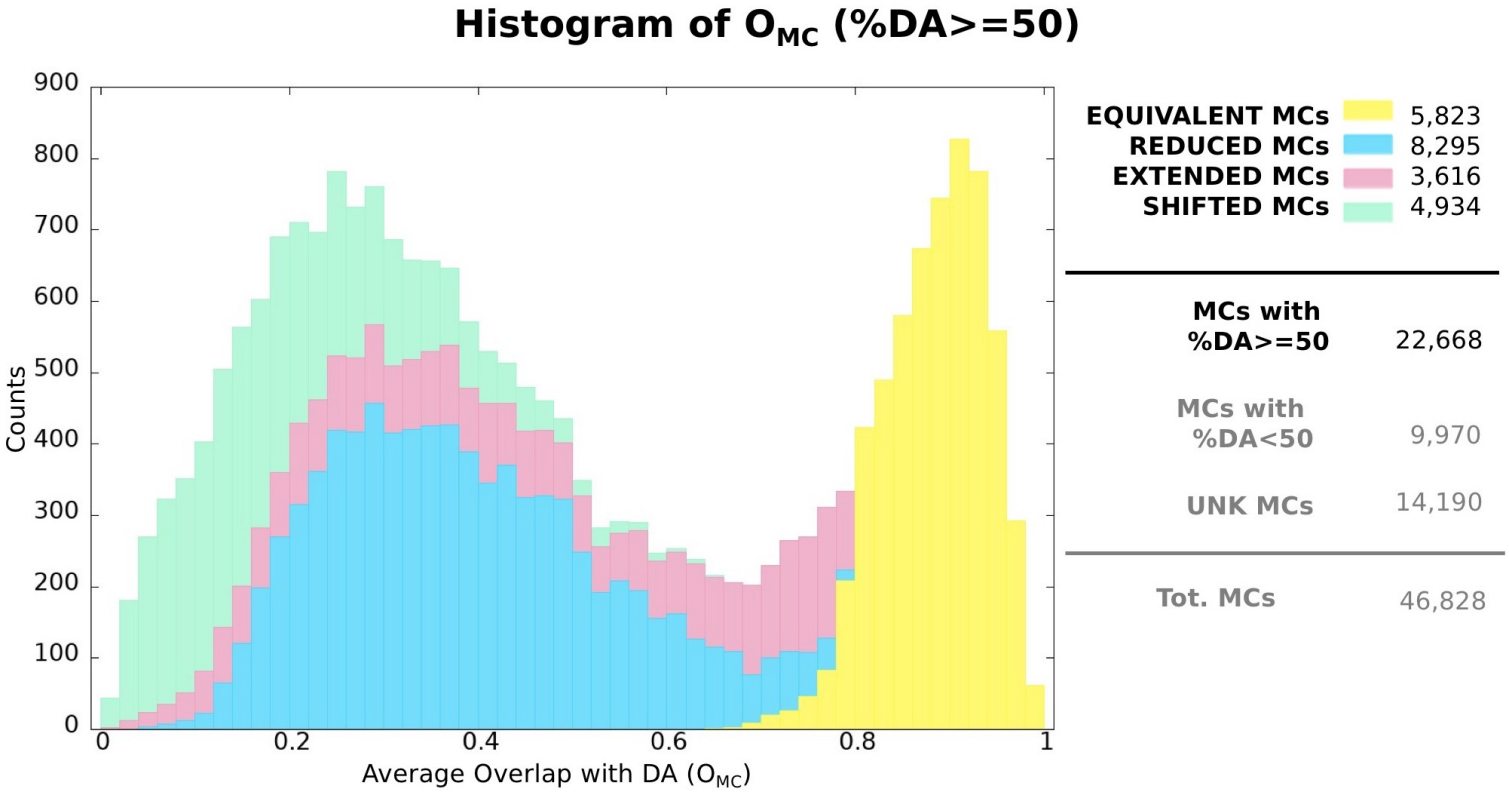
Histogram showing average overlap (*O*_*MC*_) between MCs (only those with %DA≥50) and their associated Pfam DAs. Colors reflect the contribution of each MC category to each bin (equivalent, reduced, extended and shifted, see S1(B) Fig and Methods for definitions). The legend on the right side of the histogram reports total counts of MCs in each category and, additionally, total count of MCs with %DA<50 and total count of unknown MCs.

Next, we ask to what extent boundaries of MCs and their associated Pfam families differ when evaluated on the same protein sequences. For this purpose, we consider only MCs such that %DA≥ 50 (22,668 total, or 69.5% of those with a DA annotation), that is, those for which we can perform boundary comparison on the majority of their seed sequence regions. Most of these MCs (92.5%) display a single-family DA. In Fig 4, we show the distribution of the average overlap between those seed sequences that have an exact match to the DA (DA seed sequences) and the corresponding DA annotations (see Methods for definitions). 25.7% of these MCs are classified as “equivalent”, thus having a generally good agreement with the Pfam DA’s boundaries; 36.6% are classified as “reduced” (cover only a fraction of the DA) and 16.0% as “extended” (extend significantly beyond the DA), with an accordingly smaller average overlap. Finally, 21.8% of MCs with %DA≥ 50 are classified as “shifted”. Note that while shifted MCs have poor boundary agreement with their DA annotation, the ones with very small overlaps may actually represent novel families located N-terminal or C-terminal to the DA.

We will see that the rather large number of reduced and extended MCs can, to a good extent, be explained by MC “redundancy” or the fact that oftentimes multiple MCs map to the same Pfam families while covering different parts of it. This will be discussed in detail in the next section.

To inspect the relationship between Pfam and DPCfam profile-HMMs’ hits, we first assign an equivalent label to all possible MCs irrespective of their %DA. We count 5,519 equivalent MCs with a single family-DA (see S4 Fig). Within this set, we see a strong agreement in terms of the protein hits generated by the respective profile-HMM models in Pfam family-equivalent MC pairs, suggesting that equivalent MCs reproduce to a good degree their corresponding Pfam families both in terms of their boundaries and of the area of the sequence space that they cover.

### 3.4 MC redundancy with respect to the Pfam annotation

When looking at Fig 4, we need to keep in mind that many MCs map to Pfam families that are already covered by other MCs. We call this property ‘redundancy’ and distinguish between MC pairs that are fully redundant (same DA) and those that are partially redundant (their DAs have at least one family in common) with respect to their Pfam annotation. We consider the 22,668 MCs for which %DA≥50. Among these, 73.6% have at least one fully redundant companion and 30.3% at least one partially redundant companion, with a total of 81.6% falling in at least one of the two categories. In the following, we focus on the larger set of fully redundant MCs.

In S5 Fig, we show the number of MCs as a function of their degree of redundancy (RDegree; degree 1 = no other MC carries the same DA, 2 = one other MC carries the same DA and so on). Of all pairs composed of fully redundant MCs, 30.9% are found in a parent-child relationship (see Methods), and only about 3% of these pairs are such that both MCs are simultaneously parent and child with respect to each other, reflecting a clan-like relationship. We note that in parent-child pairs at least one of the two MCs is likely to be reduced or extended with respect to the Pfam DA they share.

**Fig 5:**
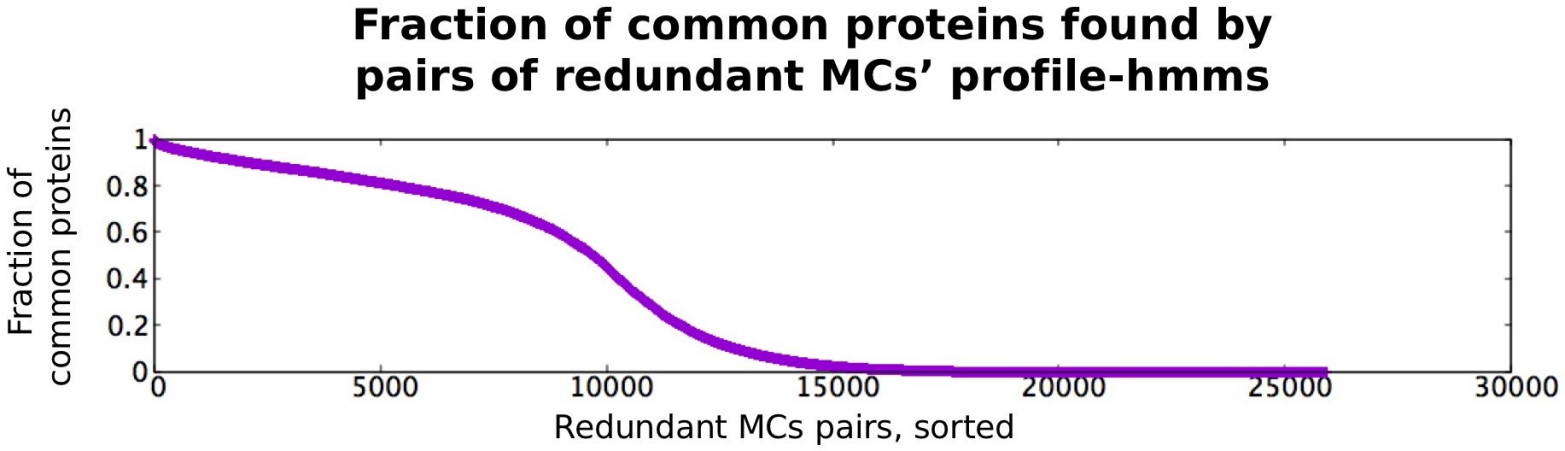
Comparison between areas of sequence space covered by MCs in fully redundant pairs. Note that we exclude MC pairs in parent-child relationships and are left with 25,980 pairs overall. For each MC, we generate the list of IDs of all proteins that map to at least one MC member (using the profile-HMM-based definition of MC membership, see Methods). Then, for each pair, we calculate the fraction of protein IDs that are shared between the two MCs (where the fraction is calculated with respect to the MC with the shorter protein ID list).

In Fig 5, we further explore the relationship between fully redundant MC pairs that are not in a parent-child correspondence. We report the fraction of proteins that are covered by both MCs (using all hits from their associated profile-HMMs) and show that 49.3% of them have less than 10% of proteins in common and 31.8% have no protein in common at all. This means that in a sizable fraction of cases, MCs in a fully redundant pair cover different portions of the protein-sequence space with the same Pfam annotation. They thus appear to represent sub-families of the associated Pfam annotation.

### 3.5 Coverage of Pfam families by DPCfam MCs

We now ask how many Pfam families out of a total of 18,198 (Pfam version 33.0) have a DPCfam’s MC counterpart. First, we investigate coverage of Pfam families by MCs regardless of how good is their boundary agreement.

**Fig 6:**
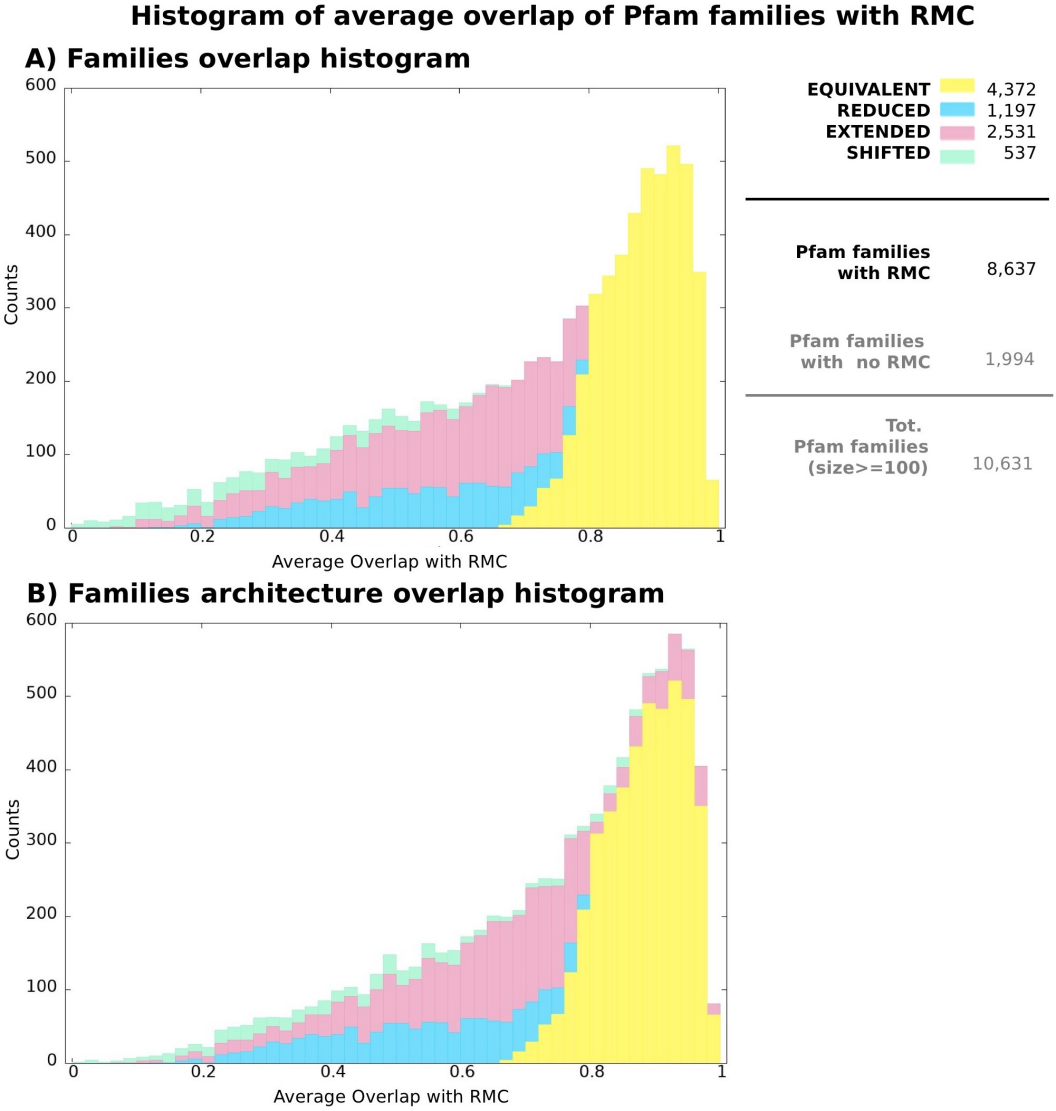
Histograms showing average overlap between Pfam families and their representative MCs. Colors reflect the contribution of each MC category to each bin (equivalent, reduced, extended and shifted, see S1(B) Fig and Methods for definitions). **A**: Overlap between individual Pfam families and their representative MCs **B**: Overlap between individual Pfam families or architectures and representative MCs. Given a Pfam family and its representative MC (same pairs as in A), we search for a better overlap of the representative MC with any multi-family architecture featuring the original Pfam family and up to two additional families. The reported average overlap value is thus the best between the overlap with the original family and any other such Pfam architecture. Note that the Pfam architecture labels (equivalent/reduced/extended/shifted) are still assigned according to the representative MC overlap to the original Pfam family so as to show to which extent the overlap in each MC category increases with respect to A).

For the sake of this comparison, we consider only Pfam families with at least 100 sequence members in UniRef50 ∩ UniprotKB (10,631 families or 58.4% of the total). For 81.3% of these families (8,647 total), we can define a representative MC (see Methods). For each representative MC, we determine whether it is equivalent, reduced, extended or shifted with respect to its corresponding Pfam family (note: the latter may not coincide with the MC’s DA). In Fig 6A, we plot the average overlap between Pfam families and their corresponding representative MCs (that is, average overlap between their member regions) and color the Pfam families according to their representative MC overlap category. In particular, 50.6% (4,372) of these families have an equivalent representative (good agreement between Pfam and representative MC boundaries), 13.8% (1,197) a reduced representative (MC covers a sub-region of the family), 29.3% (2,531) an extended representative (MC extends beyond the boundaries of the family) and, finally, 6,2% (537) a shifted representative. We further investigate the possibility that extended representatives may in fact correspond to MCs that cover a multi-family Pfam architecture (that is, with a multi-family DA). For each Pfam family in Fig 6A, we find the family architecture that has the best overlap to the representative MC (this architecture can contain either only the original family or the original family plus up to two additional ones). It can be seen, Fig 6B, that the average overlap with several representative MCs that were previously classified as extended (pink bars) increases, thus confirming that a significant fraction of these representative MCs match multi-family Pfam architectures.

Next, we compare the overall UniRef50 (v. 2017_07) protein coverage of Pfam families with that one of DPCfam MCs. For this purpose, we use hits from the profile-HMMs associated to all Pfam families (18,189 families in total) and to all MCs with average length ≥ 50 and seed size ≥ 50 (46,828 MCs total) (Methods). Pfam families and MCs cover 50.0% and 52.5% of proteins in UniRef50, respectively, with 41.1% of UniRef50 proteins being covered both by Pfam and DPCfam models. UniRef50 coverage at the residue level is 33.6% for Pfam and 46.9% for MCs. Note that Pfam coverage is much lower than the one reported for the whole of UniProtKB (77.0% of proteins, 53.2% of residues [9]). This, however, should be expected given that, compared to UniRef50, UniProtKB should contain a larger fraction of redundant proteins belonging to the largest families represented in Pfam. We note that although MC profile-HMMs outnumber Pfam families profile-HMMs about 2.6 to 1, the difference in UniRef50 protein coverage between the two classifications is only about 2.5%. This can have several explanations. First, as seen in the previous section many MCs are redundant (that is, they map to the same Pfam families). Second, the portion of UniRef50 that is still not covered by Pfam is likely to be constituted by a large number of families with a small taxonomic range [9, 36]. As a consequence, even a significant increase in the number of families may not translate into much larger coverage levels. This may also help explain why about 40% of all sequences in UniRef50 remain uncovered by either classification. In this context, it should be remembered that our analysis has been limited to DPCfam’s MCs that had length and seed size higher than 50. It is thus conceivable that the about 165K MCs that we have not taken into consideration contain a large number of small-size families that could make up at least some of the missing coverage. We briefly discuss smaller MCs in the section entitled “Small Metaclusters and DPCfam-B database” below. The rest of the unannotated sequence space may be represented by families of extremely limited taxonomic range or by compositionally-biased regions that are notoriously difficult to classify into homologous families.

### 3.6 Coverage of structure-based ECOD families by DPCfam MCs

**Fig 7:**
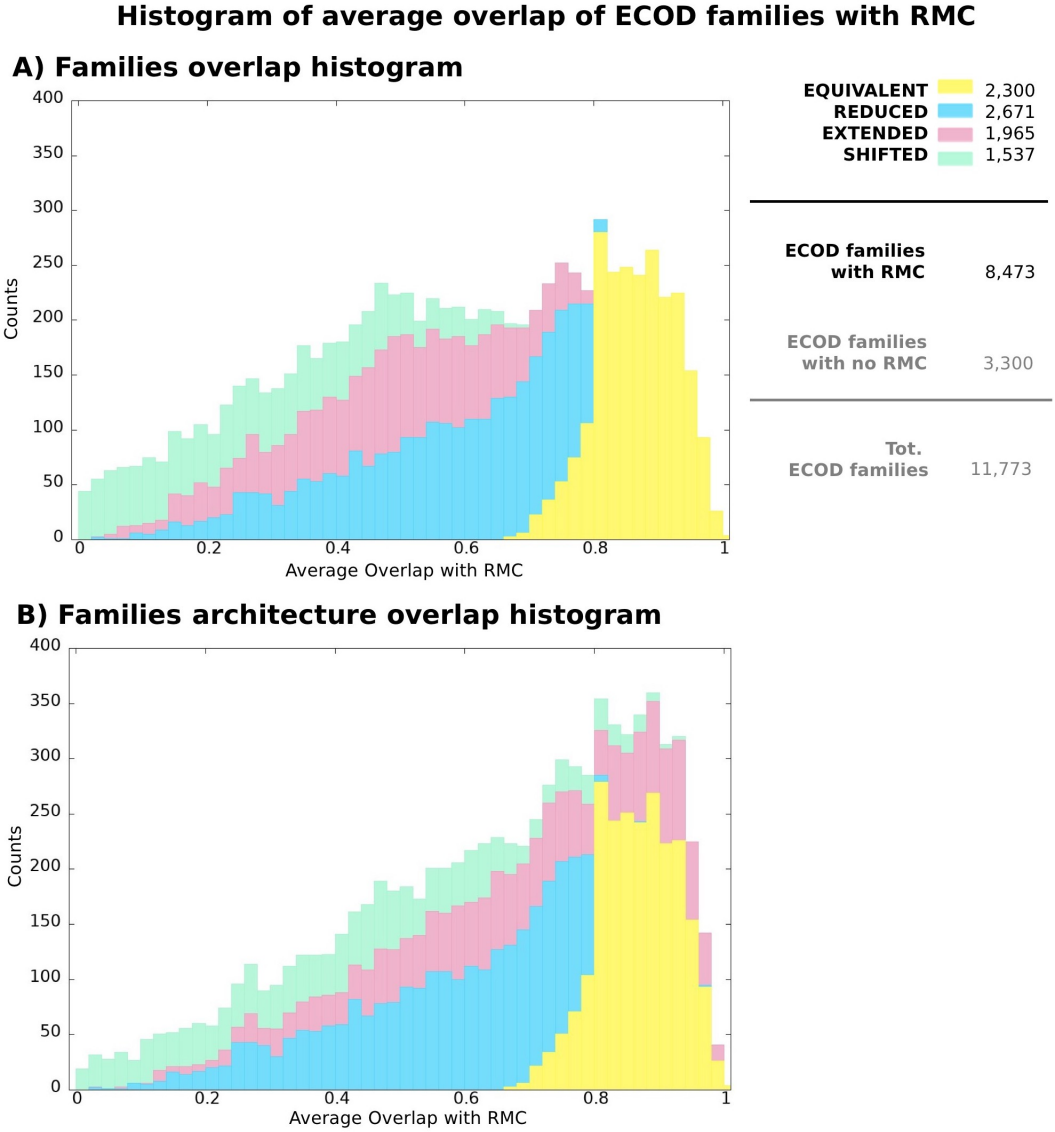
Histograms showing average overlap between ECOD families and their representative MCs. Colors reflect the contribution of each MC category to each bin (equivalent, reduced, extended and shifted, see S1(B) Fig and Methods for definitions). **A**: Overlap between individual ECOD families and their representative MCs (cfr. with Fig 6) **B**: Overlap between individual ECOD families or architectures and representative MCs. Given an ECOD family and its representative MC (same pairs as in A), we search for a better overlap of the representative MC with any multi-family architecture featuring the original ECOD family and up to two additional families. The reported average overlap value is thus the best between the overlap with the original family and any other such ECOD architecture. Note that the ECOD architecture labels (equivalent/reduced/extended/shifted) are still assigned according to the representative MC overlap to the original ECOD family so as to show to which extent the overlap in each MC category increases with respect to A).

We now compare DPCfam’s MCs to the structure-based family annotation provided by the ECOD database (Methods). ECOD splits proteins of known structure into structural domains and then clusters the domains at different levels of sequence and/or structural similarity. The specific value of comparing DPCfam with ECOD is that, although the ECOD classification is limited to families that have at least one representative of known structure, in general structure-based ECOD family boundaries should be of higher quality when evaluated against corresponding Pfam family boundaries. This is unless the Pfam family has also been built (or re-built) taking advantage of available structural information, which may well be the case. Overall, for 72.0% of ECOD families (8,473 of all 11,773) we can define a representative MC. In Fig 7A, similar to what done for Pfam, we report the overlap between ECOD families and their representative MCs. 27.1% (2,300), 31.5% (2,671), 23.2% (1,965), 18.2% (1,537) of families have an equivalent, reduced, extended, shifted representative, respectively. We can see that the percentage of ECOD-equivalent MCs is about half the percentage of Pfam-equivalent MCs. This clearly reflects the different nature of ECOD and Pfam whereby Pfam, similar to DPCfam, mainly relies on sequence conservation signals to establish family boundaries, while ECOD uses structural information.

Next, similar to what done when analysing Pfam coverage, we search for a better overlap of the representative MCs with any multi-family architecture featuring the original ECOD family and up to two additional families. Again, it can be clearly seen that many ECOD families with an extended representative are part of a larger multi-family architecture with a better overlap to the MC. Indeed, about 41% of ECOD multi-family architectures feature a representative MC with overlap > 0.75.

Finally, for comparison, we calculate the overlap between ECOD and Pfam families (with Pfam in the role played by DPCfam in the previous analysis) (S7 Fig). For the sake of this comparison, we exclude Pfam families of small size (less than 100 members in UniRef50). About 65.0% of all ECOD families have a representative among medium to large size Pfam family (or a representative PF, analogous to the previously defined representative MC). Of these, 36.2%, 33.1%, 18.3%, 12.4% are equivalent, reduced, extended, shifted, respectively. When considering ECOD multi-family architectures, 48.8% of them have a Pfam representative with overlap> 0.75.

### 3.7 MCs ‘unknown’ to Pfam

In the previous sections, we used Pfam to evaluate the overall quality of DPCfam metaclusters. For 14,190 unknown MCs (almost 30% of the total) such comparison is not possible given that none of their member regions feature a Pfam annotation. A further 9,971 MCs are partly-known MCs or MCs with a Pfam dominant architecture present in less than 50% of their seed sequences. While the latter may provide scope for Pfam family or clan extension, in this section we focus on the 14,190 unknown MCs. We first compare the percentages of low complexity, coiled-coil and disordered MCs in the unknown set versus the equivalent set (see Fig 4, yellow bars), which is used here as high-quality MC reference. We observe that the vast majority of unknown MCs (78.6%) are likely to represent structured regions of the sequence space, which constitute the traditional targets for family building, while the corresponding percentage for “equivalent” MCs is 86.4% (it was 83.5% across all MCs) (S8 Fig). This analysis supports the idea that most unknown MCs have the potential to be used as seeds for defining novel protein families. To further test this hypothesis, we teamed up with the Pfam database.

Pfam has now started using the list of unknown MCs to seed novel families and its release 35.0 already includes 63 such entries (see S2 Table). Pfam has expended a huge amount of effort to cover proteins which have known function. Thus, it is not surprising that the majority of new families added to Pfam from DPCfam so far are Domains of Unknown Function. Of the 63 added to date 42 are DUF families. However, there is a lot of value in creating these families, because they provide a target for molecular biologists to investigate and enable high throughput computational studies of complete genomes. Among the non-DUF families, a nice example is the Crinkler domain (PF20147 based on DPCfam:MC202620). This entry represents the N-terminal ubiquitin-like domain of a large family of fungal effectors. It currently matches over 3,000 proteins in the UniProt Reference Proteomes. Important plant pathogens such as Phytophthora contains more than a hundred copies of these proteins. As well as being found in plant pathogens Crinkler family proteins can be found in Arbuscular mycorrhizal fungus *Rhizophagus irregularis* [37]. The crinkler protein appears to be important for the mediation of the plant fungal symbiosis. Thus, these proteins may have important roles in agriculture. The addition of this family to Pfam enables the identification of a large class of fungal effectors that were previously not identified in the sequence databases such as UniProt, and will enable computational analysis into the function and evolution of this large class of important proteins. A second example is the N-terminal domain found in the nematode NRF6 protein (PF20146 based on DPCfam:MC15137). This domain is predicted to be extracellularly posed over a transmembrane embedded Acyltransferase domain [38]. *Caenorhabditis elegans* and *Drosophila melanogaster* both have 18 proteins containing this domain. Surprisingly, there is no protein in PDB which shows structural similarity to this domain according to Foldseek [39]. Thus, it may represent a novel protein fold. Given the role of NRF6 in binding to Fluoxetine (Prozac) it seems likely that this domain is involved in binding to a range of xenobiotics in animals and may be involved in chemosensation of toxic chemicals. Although mouse, rat and even macaque have these proteins there appears to be no human homologue, suggesting whatever the function of NRF6 and its homologues is, this function has been lost in the human lineage. We expect many more unknown MCs to be turned into Pfam families in the future.

Further, the publication of the AlphaFold database of predicted protein structures [33, 34] allows to add an extra dimension to the annotation of our UNK MCs. In particular, if a representative structure for an MC can be found in AlphaFold DB, it may provide valuable insight for determining if the MC represents a structural domain or a conserved unstructured region, whether or not the MC boundaries need to be extended or trimmed and, possibly, its functional annotation. With these goals in mind, we have thus run DPCfam MCs’ profile-HMMs against AlphaFold DB. This allowed us identifying a representative predicted structure (see Methods) for 64.6% of UNK MCs and we observe that for 56.3% of UNK MCs the AlphaFold DB representative covers at least half of the profile-HMM (the corresponding percentages for all MCs are 82.5% and 75.3%, respectively). We show a few examples of the mapping between UNK MCs and their representative AlphaFold structures in S10–S14 Figs. In particular, we select the top 25 unknown MCs in terms of the number of their seed sequences along with a few more that are not in the top list but were among those used to seed novel Pfam families. The list of the top 25 MCs with information about their inclusion in Pfam and their alignment to their representative AlphaFold structure (if present) is shown in S3 Table. For S10–S13 Figs, we select the TOP MCs that are almost completely covered by their representative structure (coverage ≥0.9 see S3 Table). In several instances, MCs appear to map rather well to what can intuitively be recognised as individual structural domains within the context of the larger structure. These include, but are not limited to, the Crinkler and NRF domains (S11(B) and S11(C) Fig). In one case (MC186369, S10(A) Fig), the MC extends over what has been predicted by AlphaFold mostly as an unstructured region. Sequences part of the Pfam family that has been built from this MC (PF20150 or 2EXR) include two conserved ExR motifs that might be functionally important. Finally, in a few cases, the MCs appear to represent small portions of larger domains (for example, S12(A) and S12(B) Fig) or to cover full length proteins, irrespective of the presence of multiple domains (S13(D) Fig and, possibly, S13(C) Fig). While these examples are unlikely to be representative of the majority of UNK MCs, they do seem to suggest that this set is rich in material for building new families and that UNK MCs can map reasonably well to structured (or, occasionally, unstructured) domains.

### 3.8 Small Metaclusters and DPCfam-B database

As stated at the beginning of the Results section, we focused our analysis on MCs with 50 or more seed sequences. However, also smaller MCs may provide useful information. In this section, we consider MCs with 25≤N<50 seed sequences and average length ≥50 aa. In total, this accounts for 34,556 additional MCs. As a first analysis step, we compute the percentage of MCs that can be assigned a Low Complexity, Disorder, Coiled Coil or Transmembrane label (according to the criteria specified in our Methods section). Overall, 41.6% of MCs can be assigned one or more of these labels. None could instead be labeled as Transmembrane. This can be compared to the case of MCs of larger size (≥50 seed sequences), where labels of Low Complexity, Disorder or Coiled Coil could be assigned to 16.5% of MCs (Fig 3B) and a Transmembrane label to 3.5% of MCs. Next, we compare small MCs to Pfam annotations. Of 34,556 small MCs, 20.5% (9,878) display a %DA≥50, of which 1,153 are “equivalent” MCs, 3,619 are “reduced”, 1,664 are “extended” and 3,442 are “shifted”. In S9 Fig we show the average overlap histogram with their DA for these MCs (cfr. Fig 4 for larger MCs). Finally, 18,989 MCs have no Pfam annotation (unknown MCs). Overall these results suggest that, while this set of MCs should be on average of lower quality than the one constituted of MCs of larger size, as a whole it is likely to contain quite valuable information. We therefore decided to publish also these smaller MCs, albeit as a separate database that we name DPCfam-B.

## 4 Discussion and conclusions

Most leading protein family databases take advantage of some level of manual curation in the definition of their families. However, curated annotation is becoming a considerable burden as the number of proteins for which a sequence is available skyrockets. In this context, automatic protein family classification could represent a tool for rapid, high-level analysis of big, largely unannotated databases such as those produced by metagenomics projects. At the same time, it could assist in the extension of the more accurate, manually supervised classification schemes.

The idea of combining large protein databases, sequence alignments and clustering to produce family classification schemes is of course not new (e.g. MMseqs2 [15], COG, [40] etc.). However, most automatic methods have focused on clustering of full-length protein sequences. The few methods that, to the best of our knowledge, have attempted to automatically classify evolutionary modules in proteins (most typically representing structural domains) (ADDA [13], EVEREST [14]), have faced scalability and/or maintenance problems.

In this work, we describe the application of our method DPCfam to the automatic family annotation of UniRef50, a database including ∼ 23,000,000 sequences. DPCfam uses local sequence alignments (BLAST) and takes advantage of the power and efficiency of Density Peak Clustering [17] to split proteins into separate evolutionary modules. The method itself was introduced previously in [16] on a much smaller dataset. The most important technical contribution of this work is an efficient and scalable implementation of the algorithm. The size of the clustering problem which we solve here is enormous: it involves estimating the distances between $O(10^{16})$ pairs of sequence fragments. For this reason, our pipeline is implemented to run on HPC facilities; in particular, it requires at least one node with a RAM memory of ∼ 0.5 Tb, better if 1 Tb. In S1 Table we report the computational costs for each step of the method. DPCfam is structured in a way that allows for small incremental updates of the metaclusters at relatively low computational cost. When adding new sequences to the database it is not necessary to compute from scratch the distance matrix, namely the most demanding step of the pipeline (without taking the generation of sequence alignments into account), which can instead be updated thus dramatically reducing running times. This can be achieved using an approximation whereby new primary clusters are generated from alignments of the novel protein sequences to the full, updated protein database, while previous primary clusters are left untouched. An increment of 10% in the underlying protein sequence database will cost approximately 14% of the computational time required by the original run. If the underlying sequence database becomes significantly larger than the original one, however, this approximation becomes too rough and updating the database would require a complete rerun of the protocol.

The analysis of UniRef50 (v. 2017_07) that we present here produces > 45, 000 well-populated metaclusters (MCs). The majority of these MCs exhibit at least some overlap with families in the Pfam classification, which we use here as our main manually-curated reference. We have shown that the DPCfam-generated classification of the protein sequence space produces MCs that for the vast majority appear to be sound in terms of the homologous relationships that can be inferred to exist between their seed sequences. About half of MCs are in clear correspondence to one or more Pfam families (%DA≥ 50). Among these, boundary agreement with the corresponding families vary, with roughly 25% being classified as “equivalent” or very close to Pfam boundaries’ definition. We note that while the thousands of equivalent MCs are proof that DPCfam can produce good-quality families in an unsupervised manner, “extended” and “reduced” MCs are arguably of higher interest for follow-up work. Indeed, as shown by a number of examples discussed in our previous work [16], MCs in these two categories may represent opportunities for improving existing classifications. Likely the most important contribution of our work to protein family classification lies, however, in the subset of about 14,000 unknown MCs, for which no seed sequence could be found to have a Pfam annotation. Indeed, preliminary analysis performed in collaboration with the Pfam database suggests that a significant fraction of these MCs might be turned into novel families with minimal manual intervention. This highlights the value of DPCfam both for further expansion of existing family classifications and, potentially, for large-scale annotation of newly-sequenced, sparsely-characterised protein datasets such as those generated by metagenomics projects.

There are some aspects of the DPCfam pipeline that we plan to revisit and improve in the future.

By far the most important computational bottleneck of the whole procedure is constituted by the all-versus-all sequence alignment searches. In this work we have used the alignment program BLAST, however, faster alternatives may be available (e.g., lastal [41], DIAMOND [42], MMseqs2 [15]).

Further, as discussed in the Results section, we observe a rather high degree of redundancy among the DPCfam-generated MCs. Some of these redundancies might be resolved by applying more aggressive metaclustering and/or merging approaches. At the same time, redundancy can carry important information about family and domain boundaries, which could be of great value especially when analysing MCs that lack annotation. We show one interesting example of two redundant MCs and their relationship to structural domains of the Calmodulin binding protein-like Pfam family in S6 Fig. Also, we have shown that many redundant MC pairs map to largely separate sets of proteins. As such they can be considered part of MC clan-like structures. In the future, we plan to further investigate the evolutionary signal behind these structures, for example whether the splits occur along different phylogenetic clade boundaries or, potentially, sub-functional groups.

Finally, since it is clear that a detailed analysis of the entire set of more than 45,000 MCs generated by DPCfam will require a lot of additional work, we make all relevant data publicly available, so that also other researchers have the possibility to take advantage of them. We do this in two different ways. First, we deposit the DPCfam classification in a public data repository (https://doi.org/10.5281/zenodo.6900559) where for each MC we provide the list of its seed sequences and their associated profile-HMM; also, we provide xml tables with summary information about MCs such as size, average length, Pfam annotation, etc. We also provide xml tables of UniRef50 proteins (v. 2017_07) with Pfam and DPCfam annotation. Second, we built an ad-hoc DPCfam website (https://dpcfam.areasciencepark.it/). The website accepts queries in the form of MC, Pfam family or protein IDs. Similar to the Zenodo xml files, MC webpages report information about size, average length and relationships with Pfam families, together with the complete list of MC seed sequences and, when present, links to representative AlphaFold DB v2 structures. Also, MC seed sequences can be visualised in the context of the full-length proteins they are part of together with other MCs and Pfam families that may additionally map to it. Multiple sequence alignments and profile-HMMs of all MCs are readily available for download. In the near future, we aim to broaden the scope of the website by including a wider range of annotations (such as phylogenetic information) both at the MC and at the protein level and, potentially, by adding protein-search features.

## Supporting information

S1 Table Approximate computational times, minimum RAM requirements and I/O charge of the DPCfam method steps. We report only minimum RAM requirements that cannot be met on a common workstation.
(PDF)

S2 Table List of new families in Pfam version 35.0 derived from DPCfam unknown MCs. (PDF)

S3 Table List of TOP 25 largest UNK MCs with information about their inclusion in Pfam and their alignment to their representative AlphaFold structure, when present. (PDF)

S1 FigGraphical representation of A) the overlapping regions between an MC seed sequence S (blue) and the part of the same protein sequence covered by its DA P (orange), which are then used to compute the quantities *F*_*red*_ and *F*_*ext*_ and B) the four categories of metaclusters the definition of which is based on *F*_*red*_ and *F*_*ext*_ averaged over all seed sequence-DA pairs in a cluster ($F_{ext}^{MC}$ and $F_{red}^{MC}$, respectively).See Methods in main text.(TIF)

S2 Fig Example of a disordered MC (MC122787) with a well-conserved multiple sequence alignment. MC122787 has 41% of amino-acids in a disordered region and it is classified as an unknown MC, that is, none of its seed sequences carries a Pfam annotation. A) MC and Pfam family architecture of DNA-binding protein F1LX23, a representative of seed sequence of MC12287. The thin black like represents the full length protein sequence. Boxes above the sequence represent DPCfam MC annotations: MC434132 from aa 22 to aa 55 (pink), MC122787 from aa 74 to 246 (blue), MC181414 from aa 479 to 678 (green); boxes below the sequence represent instead Pfam family annotations: PF01352 (KRAB) from aa 3 to 43 (green), 13 repeats of PF00096 (zf-C2H2) from aa 395 to 753 (purple) (the image is generated by the DPCfam website, https://dpcfam.areasciencepark.it/). B) Three-dimensional structure of protein F1LX23 as predicted by AlphaFold2 (Cramer, Patrick. “AlphaFold2 and the future of structural biology.” Nature Structural & Molecular Biology 28(9) (2021): 704–705.). Colors indicate where DPCfam annotations map onto the protein structure (color scheme is the same as the one used in A) for MCs). It can be seen that MC12287 (blue) is predicted to be in an unstructured region. C) MC122787’s multiple sequence alignment with conservation, quality, consensus and occupancy histograms. Note that while only a few representative sequences are shown, conservation and consensus are calculated on the full set of aligned sequences. Figure drawn with Jalview (Waterhouse AM, Procter JB, Martin DMA, Clamp M, Barton GJ “Jalview Version 2-a multiple sequence alignment editor and analysis workbench”. Bioinformatics 25 (2009): 1189–1191.).
(TIF)

S3 Fig Size of MCs (purple) and Pfam families (green), ranked from the largest (left) to the smallest (right). MC size is calculated as the number of seed sequences. Pfam families (v. 32) are all those in Uniref50 ∩ UniprotKB (18,189 total) and family size is the ‘full’ size, that is, not only considering Pfam seed sequences. The blue horizontal line corresponds to a size of 50; MCs smaller than 50 have not been considered in the analysis presented in this work.
(TIF)

S4 Fig Comparison between profile-HMMs from equivalent DPCfam MC-Pfam family pairs; note that in this case, equivalent MCs with a single family-DA are considered irrespective of their %DA (5,519 MCs in total). The red curve shows the fraction of common protein hits between the profile-HMM models in each pair (running average, window of size 50), note that in this case we compare protein IDs with no specific requirement for overlap between hits to the same ID; the other curves represent the fraction of protein hits that are found only by the DPCfam (green) or the Pfam (blue) models, respectively (running averages as above). For each MC-Pfam family pair, fractions are computed with respect to the union of all protein hits found by the MC and the Pfam model. Pairs along the x-axis are sorted by decreasing fraction of common hits. About 60% of profile-HMMs from equivalent MC-family pairs share more than 80% of their UniRef50 hits and about 80% of them share more than half of their hits. For around 9.7% of equivalent profile-HMMs, the fraction of shared hits is instead less than 25%. On average, these latter profile-HMMs appear to represent large, diverse families, for which DPCfam and Pfam tend to capture different member regions. In fact, the average size of Pfam families sharing less than 25% of hits with their associated equivalent MC is 2,562, this is to be compared to an overall average size of 800.
(TIF)

S5 Fig We calculate the number of Dominant Architectures (DA in the main text) that map to a single MC (degree = 1) and of those that instead map to multiple MCs (degree = 2, 3, etc.) (y axis in logarithmic scale, base 10). (TIF)

S6 Fig Example of a redundant MCs (MC408780) providing information about the structural features of a Pfam family. **A**: location of a SSR from MC220539 (pink), a SSRs from MC408780 (blue) and Pfam family PF07887 (pale-blue), on protein T1MG69. MC220539 and MC408780 both feature PF07887 (Calmodulin binding protein-like) as DA, the former with “equivalent” boundaries, the latter with “reduced” boundaries. **B**: structural prediction of PF07887 by trRosetta (from the Pfam website), with highlighted in blue the position of the “reduced” MC (MC408780), as found by running the MC profile-HMM with HHpred (standard parameters) against the T1MG69 sequence. As noted in the Pfam blog-post “Folding the protein universe” (31 march 2021, see https://xfam.wordpress.com/2021/03/03/folding-the-protein-universe/), structural prediction of the Calmodulin binding protein-like family shows three separate structural domains (I, II and III approximately). Indeed, in Pfam version 35 this family has been split in three different families. It can be seen that two of these domains have close correspondence to the “reduced” MC (MC408780).
(TIF)

S7 Fig Histogram of ECOD families average overlap with their representative Pfam Families (RPFs, see Methods for definition). Columns are colored according to the RPF boundary classification (equivalent, reduced, extended and shifted). **A**: Overlap between individual ECOD families and their RPF. **B**: Overlap between individual ECOD families or architectures and RPFs. Given an ECOD family and its RPF (same pairs as in A), we search for a better overlap of the RPF with any multi-family architecture featuring the original ECOD family and up to two additional ECOD families. The reported average overlap value is thus the best between the overlap with the original family and any other such ECOD architecture. Note that the ECOD architecture labels (equivalent/reduced/extended/shifted) are still assigned according to the RPF overlap to the original ECOD family so as to show to which extent the overlap in each ECOD family category increases with respect to A).
(TIF)

S8 Fig Venn diagrams showing the percentage of Low Complexity MCs (green), Disordered MCs (red) and Coiled-Coil MCs (purple), respectively (see Methods for definitions) in A) Equivalent MCs (equivalent to a Pfam family, Methods). Panel B: MCs unknown to Pfam. 86.4% and 78.6% is the number of MCs featuring none of the above regions among equivalent and unknown MCs, respectively.
(TIF)

S9 Fig Histogram showing average overlap (*O*_*MC*_) between “small” MCs (25 ≤ *N* < 50 SSRs, %DAF≥50) and their associated Pfam DAs. Colors reflect the contribution of each MC category to each bin (equivalent, reduced, extended and shifted, see also S1B Fig). The legend on the right side of the histogram reports total counts of MCs in each category, total count of MCs with %DA< 50 and total count of unknown MCs.
(TIF)

S10 Fig Examples of mapping of MC to their representative AlphaFold structure. The part of the sequence of the AlphaFold protein that aligns to the MC profile-HMM is shown in orange. Above each example we report the ID of the AlphaFold structure, the MC number, the Pfam family that was build from the MC (if any) and the position of the MC in the list of largest unknown MCs (S3 Table). Molecular graphics and analyses performed with UCSF Chimera (Pettersen EF, Goddard TD, Huang CC, Couch GS, Greenblatt DM, Meng EC, Ferrin TE. “UCSF Chimera–a visualization system for exploratory research and analysis.” J Comput Chem. 25 (2004):1605–12).
(TIF)

S11 Fig Examples of mapping of MC to their representative AlphaFold structure. The part of the sequence of the AlphaFold protein that aligns to the MC profile-HMM is shown in orange. Above each example we report the ID of the AlphaFold structure, the MC number, the Pfam family that was build from the MC (if any) and the position of the MC in the list of largest unknown MCs (S3 Table). Molecular graphics and analyses performed with UCSF Chimera (Pettersen EF, Goddard TD, Huang CC, Couch GS, Greenblatt DM, Meng EC, Ferrin TE. “UCSF Chimera–a visualization system for exploratory research and analysis.” J Comput Chem. 25 (2004):1605–12).
(TIF)

S12 Fig Examples of mapping of MC to their representative AlphaFold structure. The part of the sequence of the AlphaFold protein that aligns to the MC profile-HMM is shown in orange. Above each example we report the ID of the AlphaFold structure, the MC number, the Pfam family that was build from the MC (if any) and the position of the MC in the list of largest unknown MCs (S3 Table). Molecular graphics and analyses performed with UCSF Chimera (Pettersen EF, Goddard TD, Huang CC, Couch GS, Greenblatt DM, Meng EC, Ferrin TE. “UCSF Chimera–a visualization system for exploratory research and analysis.” J Comput Chem. 25 (2004):1605–12).
(TIF)

S13 Fig Examples of mapping of MC to their representative AlphaFold structure. The part of the sequence of the AlphaFold protein that aligns to the MC profile-HMM is shown in orange. Above each example we report the ID of the AlphaFold structure, the MC number, the Pfam family that was build from the MC (if any) and the position of the MC in the list of largest unknown MCs (S3 Table). Molecular graphics and analyses performed with UCSF Chimera (Pettersen EF, Goddard TD, Huang CC, Couch GS, Greenblatt DM, Meng EC, Ferrin TE. “UCSF Chimera–a visualization system for exploratory research and analysis.” J Comput Chem. 25 (2004):1605–12).
(TIF)

S14 Fig Examples of mapping of MC to their representative AlphaFold structure. The part of the sequence of the AlphaFold protein that aligns to the MC profile-HMM is shown in orange. Above each example we report the ID of the AlphaFold structure, the MC number, the Pfam family that was build from the MC (if any). Molecular graphics and analyses performed with UCSF Chimera (Pettersen EF, Goddard TD, Huang CC, Couch GS, Greenblatt DM, Meng EC, Ferrin TE. “UCSF Chimera–a visualization system for exploratory research and analysis.” J Comput Chem. 25 (2004):1605–12).
(TIF)

## References

[1] UniProt: the universal protein knowledgebase in 2021. Nucleic Acids Research. 2021;49(D1):D480–D489. doi: 10.1093/nar/gkaa1100 33237286 PMC7778908

[2] Mitchell AL, Almeida A, Beracochea M, Boland M, Burgin J, Cochrane G, et al. MGnify: the microbiome analysis resource in 2020. Nucleic acids research. 2020;48(D1):D570–D578. doi: 10.1093/nar/gkz1035 31696235 PMC7145632

[3] Ponting CP, Russell RR. The natural history of protein domains. Annual review of biophysics and biomolecular structure. 2002;31(1):45–71. doi: 10.1146/annurev.biophys.31.082901.134314 11988462

[4] Punta M, Ofran Y. The Rough Guide to In Silico Function Prediction, or How To Use Sequence and Structure Information To Predict Protein Function. PLOS Computational Biology. 2008;4(10):1–7. doi: 10.1371/journal.pcbi.1000160 18974821 PMC2518264

[5] Tompa P, Fuxreiter M, Oldfield CJ, Simon I, Dunker AK, Uversky VN. Close encounters of the third kind: disordered domains and the interactions of proteins. Bioessays. 2009;31(3):328–335. doi: 10.1002/bies.200800151 19260013

[6] Letunic I, Bork P. 20 years of the SMART protein domain annotation resource. NAR. 2017;46(D1):D493–D496. doi: 10.1093/nar/gkx922 PMC5753352 29040681

[7] Akiva E, et al. The Structure–Function Linkage Database. NAR. 2013;42(D1):D521–D530. doi: 10.1093/nar/gkt1130 24271399 PMC3965090

[8] Cheng H, et al. Manual classification strategies in the ECOD database. Proteins. 2015;83(7):1238–1251. doi: 10.1002/prot.24818 25917548 PMC4624060

[9] Mistry J, Chuguransky S, Williams L, Qureshi M, Salazar G, Sonnhammer ELL, et al. Pfam: The protein families database in 2021. Nucleic Acids Research. 2020;49(D1):D412–D419. doi: 10.1093/nar/gkaa913 PMC7779014 33125078

[10] Mitchell AL, et al. InterPro in 2019: improving coverage, classification and access to protein sequence annotations. NAR. 2018;47(D1):D351–D360. doi: 10.1093/nar/gky1100 PMC6323941 30398656

[11] Lu S, et al. CDD/SPARCLE: the conserved domain database in 2020. NAR. 2020;48(D1):D265–D268. doi: 10.1093/nar/gkz991 31777944 PMC6943070

[12] Enright AJ, et al. An efficient algorithm for large-scale detection of protein families. NAR. 2002;30 7:1575–84. doi: 10.1093/nar/30.7.1575 11917018 PMC101833

[13] Heger A, Holm L. Exhaustive Enumeration of Protein Domain Families. JMB. 2003;328(3):749–767. doi: 10.1016/S0022-2836(03)00269-9 12706730

[14] Portugaly E, et al. EVEREST: automatic identification and classification of protein domains in all protein sequences. BMC bioinformatics. 2006;7(1):277. doi: 10.1186/1471-2105-7-277 16749920 PMC1533870

[15] Steinegger M, Söding J. MMseqs2 enables sensitive protein sequence searching for the analysis of massive data sets. Nature biotechnology. 2017;35(11):1026–1028. doi: 10.1038/nbt.3988 29035372

[16] Russo ET, Laio A, Punta M. Density Peak clustering of protein sequences associated to a Pfam clan reveals clear similarities and interesting differences with respect to manual family annotation. BMC Bioinformatics. 2021;22(1):121. doi: 10.1186/s12859-021-04013-x 33711918 PMC7955657

[17] Rodriguez A, Laio A. Clustering by fast search and find of density peaks. Science. 2014;344(6191):1492–1496. doi: 10.1126/science.1242072 24970081

[18] Altschul SF, Gish W, Miller W, Myers EW, Lipman DJ. Basic local alignment search tool. Journal of molecular biology. 1990;215(3):403–410. doi: 10.1016/S0022-2836(05)80360-2 2231712

[19] Mistry J, et al. Challenges in homology search: HMMER3 and convergent evolution of coiled-coil regions. NAR. 2013;41(12):e121–e121. doi: 10.1093/nar/gkt263 23598997 PMC3695513

[20] Forum MP. MPI: A message-passing interface standard; 1994.

[21] Davis IJ. A fast radix sort. The computer journal. 1992;35(6):636–642. doi: 10.1093/comjnl/35.6.636

[22] Arpaci-Dusseau RH, Arpaci-Dusseau AC. Operating Systems: Three Easy Pieces. 1st ed. Arpaci-Dusseau Books; 2018.

[23] Li W, Godzik A. Cd-hit: a fast program for clustering and comparing large sets of protein or nucleotide sequences. Bioinformatics. 2006;22(13):1658–1659. doi: 10.1093/bioinformatics/btl158 16731699

[24] Edgar RC. MUSCLE: multiple sequence alignment with high accuracy and high throughput. NAR. 2004;32(5):1792–1797. doi: 10.1093/nar/gkh340 15034147 PMC390337

[25] Mier P, Paladin L, Tamana S, Petrosian S, Hajdu-Soltész B, Urbanek A, et al. Disentangling the complexity of low complexity proteins. Briefings in Bioinformatics. 2019;21(2):458–472. doi: 10.1093/bib/bbz007 PMC7299295 30698641

[26] Crick F. The packing of *α*-helices: simple coiled-coils. Acta crystallographica. 1953;6(8-9):689–697. doi: 10.1107/S0365110X53001964

[27] Dyson HJ, Wright PE. Intrinsically unstructured proteins and their functions. Nature reviews Molecular cell biology. 2005;6(3):197–208. doi: 10.1038/nrm1589 15738986

[28] Tusnady GE, Simon I. Principles governing amino acid composition of integral membrane proteins: application to topology prediction. Journal of molecular biology. 1998;283(2):489–506. doi: 10.1006/jmbi.1998.2107 9769220

[29] Camacho C, Coulouris G, Avagyan V, Ma N, Papadopoulos J, Bealer K, et al. BLAST+: architecture and applications. BMC bioinformatics. 2009;10(1):421. doi: 10.1186/1471-2105-10-421 20003500 PMC2803857

[30] Ludwiczak J, Winski A, Szczepaniak K, Alva V, Dunin-Horkawicz S. DeepCoil—a fast and accurate prediction of coiled-coil domains in protein sequences. Bioinformatics. 2019;35(16):2790–2795. doi: 10.1093/bioinformatics/bty1062 30601942

[31] Mészáros B, Erdős G, Dosztányi Z. IUPred2A: context-dependent prediction of protein disorder as a function of redox state and protein binding. Nucleic acids research. 2018;46(W1):W329–W337. doi: 10.1093/nar/gky384 29860432 PMC6030935

[32] Käll L, Krogh A, Sonnhammer EL. A combined transmembrane topology and signal peptide prediction method. Journal of molecular biology. 2004;338(5):1027–1036. doi: 10.1016/j.jmb.2004.03.016 15111065

[33] Jumper J, Evans R, Pritzel A, Green T, Figurnov M, Ronneberger O, et al. Highly accurate protein structure prediction with AlphaFold. Nature. 2021;596(7873):583–589. doi: 10.1038/s41586-021-03819-2 34265844 PMC8371605

[34] Varadi M, Anyango S, Deshpande M, Nair S, Natassia C, Yordanova G, et al. AlphaFold Protein Structure Database: massively expanding the structural coverage of protein-sequence space with high-accuracy models. Nucleic acids research. 2022;50(D1):D439–D444. doi: 10.1093/nar/gkab1061 34791371 PMC8728224

[35] Qian J, Luscombe NM, Gerstein M. Protein family and fold occurrence in genomes: power-law behaviour and evolutionary model. Journal of molecular biology. 2001;313(4):673–681. doi: 10.1006/jmbi.2001.5079 11697896

[36] Mistry J, Coggill P, Eberhardt RY, Deiana A, Giansanti A, Finn RD, et al. The challenge of increasing Pfam coverage of the human proteome. Database. 2013;2013. doi: 10.1093/database/bat023 23603847 PMC3630804

[37] Voß S, Betz R, Heidt S, Corradi N, Requena N. RiCRN1, a crinkler effector from the arbuscular mycorrhizal fungus Rhizophagus irregularis, functions in arbuscule development. Frontiers in microbiology. 2018;9:2068. doi: 10.3389/fmicb.2018.02068 30233541 PMC6131194

[38] Choy RK, Thomas JH. Fluoxetine-resistant mutants in C. elegans define a novel family of transmembrane proteins. Molecular cell. 1999;4(2):143–152. doi: 10.1016/S1097-2765(00)80362-7 10488330

[39] van Kempen M, Kim S, Tumescheit C, Mirdita M, Söding J, Steinegger M. Foldseek: fast and accurate protein structure search. bioRxiv. 2022;. 10.1038/s41587-023-01773-0 PMC10869269 37156916

[40] Tatusov RL, et al. The COG database: a tool for genome-scale analysis of protein functions and evolution. NAR. 2000;28(1):33–36. doi: 10.1093/nar/28.1.33 10592175 PMC102395

[41] Kiełbasa SM, Wan R, Sato K, Horton P, Frith MC. Adaptive seeds tame genomic sequence comparison. Genome research. 2011;21(3):487–493. doi: 10.1101/gr.113985.110 21209072 PMC3044862

[42] Buchfink B, Reuter K, Drost HG. Sensitive protein alignments at tree-of-life scale using DIAMOND. Nature methods. 2021;18(4):366–368. doi: 10.1038/s41592-021-01101-x 33828273 PMC8026399

