# Supplementary material for: DPCfam: Unsupervised protein family classification by Density Peak Clustering of large sequence datasets
Source: PLoS Comput Biol. 2022 Oct 19;18(10):e1010610. doi: 10.1371/journal.pcbi.1010610 (PMC9621593; doi:10.1371/journal.pcbi.1010610)
Supplement: S1 Table — We report only minimum RAM requirements that cannot be met on a common workstation. (PDF) [file pcbi.1010610.s001.pdf]

DPCfam step	CPU hours	Min RAM requirements	Input size/format	Output size/format
**Alignment generation with blastp**	750,000	N/A	70 GB, FASTA	3.5 TB, CSV
**Primary clustering**	50,000	N/A	3.5 TB, CSV	0.5 TB, binary
**Generation of Primary clusters' Distance Matrix**	30,000	0.5 TB	0.5 TB, binary	2.5 TB, binary
**Metaclustering and merging**	8	N/A	2.5 TB, binary	7 GB, CSV
**Final output generation**	neglectable	N/A	7 GB, CSV + 0.5 TB, binary	500 MB, CSV
