# Supplementary material for: DPCfam: Unsupervised protein family classification by Density Peak Clustering of large sequence datasets
Source: PLoS Comput Biol. 2022 Oct 19;18(10):e1010610. doi: 10.1371/journal.pcbi.1010610 (PMC9621593; doi:10.1371/journal.pcbi.1010610)
Supplement: S2 Table — (PDF) [file pcbi.1010610.s002.pdf]

PfamA accession number	PfamA family name	Family Description	Seed Source
PF20146	NRF	Nose resistant-to-fluoxetine protein, N-terminal domain	DPCfam:MC15137
PF20147	Crinkler	Crinkler effector protein N-terminal domain	DPCfam:MC202620
PF20148	DUF6531	Domain of unknown function (DUF6531)	DPCfam:MC297638
PF20149	DUF6532	Domain of unknown function (DUF6532)	DPCfam:MC405686
PF20150	2EXR	2EXR family	DPCfam:MC186369
PF20151	DUF6533	Family of unknown function (DUF6533)	DPCfam:MC411630
PF20152	DUF6534	Family of unknown function (DUF6534)	DPCfam:MC304269
PF20153	DUF6535	Family of unknown function (DUF6535)	DPCfam:MC487727
PF20154	LNT_N	Apolipoprotein N-acyltransferase N-terminal domain	DPCfam:MC385670
PF20155	TMP_3	Tape measure protein	DPCfam:MC116910
PF20163	DUF6536	Family of unknown function (DUF6536)	DPCfam:MC126793
PF20167	Transposase_32	Putative plant transposon protein	DPCfam:MC463665
PF20168	PDS5	Sister chromatid cohesion protein PDS5 protein	DPCfam:MC486108
PF20169	DUF6537	Family of unknown function (DUF6537)	DPCfam:MC38141
PF20172	DUF6538	Domain of unknown function (DUF6538)	DPCfam:MC100804
PF20173	DUF6539	Family of unknown function (DUF6539)	DPCfam:MC10187
PF20174	DUF6540	Family of unknown function (DUF6540)	DPCfam:MC102650
PF20175	Tra1_central	Tra1 HEAT repeat central region	DPCfam:MC101642
PF20176	DUF6541	Family of unknown function (DUF6541)	DPCfam:MC103573
PF20177	DUF6542	Domain of unknown function (DUF6542)	DPCfam:MC107385
PF20178	DUF6543	Family of unknown function (DUF6543)	DPCfam:MC109179
PF20179	MSS51_C	MSS51 C-terminal domain	DPCfam:MC112665
PF20181	DUF6544	Family of unknown function (DUF6544)	DPCfam:MC126405
PF20182	DUF6545	Family of unknown function (DUF6545)	DPCfam:MC121555
PF20183	DUF6546	Family of unknown function (DUF6546)	DPCfam:MC186755
PF20206	Tra1_ring	Tra1 HEAT repeat ring region	DPCfam:MC101642
PF20209	DUF6570	Domain of unknown function (DUF6570)	DPCfam:MC448731
PF20210	Laa1_Sip1_HTR5	Laa1/Sip1/HEATR5 HEAT repeat region	DPCfam:MC476572

PF20220	ABC_toxin_N	ABC toxin N-terminal region	DPCfam:MC130378
PF20222	DUF6581	Family of unknown function (DUF6581)	DPCfam:MC61581
PF20231	DUF6589	Family of unknown function (DUF6589)	DPCfam:MC213898
PF20232	T6SS_FHA_C	C-terminal domain of Type VI secretion system FHA protein	DPCfam:MC139621
PF20233	DUF6590	Family of unknown function (DUF6590)	DPCfam:MC80334
PF20235	DUF6592	Family of unknown function (DUF6592)	DPCfam:MC363598
PF20236	DUF6593	Family of unknown function (DUF6593)	DPCfam:MC83679
PF20237	DUF6594	Family of unknown function (DUF6594)	DPCfam:MC126457
PF20238	DUF6595	Family of unknown function (DUF6595)	DPCfam:MC121371
PF20239	DUF6596	Family of unknown function (DUF6596)	DPCfam:MC238509
PF20240	DUF6597	Domain of unknown function (DUF6597)	DPCfam:MC439487
PF20241	DUF6598	Domain of unknown function (DUF6598)	DPCfam:MC453907
PF20243	MbnP	MbnP	DPCfam:MC490110
PF20244	DUF6599	Family of unknown function (DUF6599)	DPCfam:MC429792
PF20245	DUF6600	Family of unknown function (DUF6600)	DPCfam:MC285095
PF20246	DUF6601	Family of unknown function (DUF6601)	DPCfam:MC245544
PF20247	DUF6602	Domain of unknown function (DUF6602)	DPCfam:MC365183
PF20248	DUF6603	Family of unknown function (DUF6603)	DPCfam:MC188644
PF20249	VasX_N	VasX toxin N-terminal region	DPCfam:MC292492
PF20251	Big_14	Bacterial Ig-like domain	DPCfam:MC458669
PF20252	BIG2_C	BIG2 C-terminal domain	DPCfam:MC407994
PF20253	DUF6604	Family of unknown function (DUF6604)	DPCfam:MC324335
PF20254	DUF6605	Domain of unknown function (DUF6605)	DPCfam:MC471977
PF20255	DUF6606	Family of unknown function (DUF6606)	DPCfam:MC501646
PF20410	DUF6696	Domain of unknown function (DUF6696)	DPCfam:MC14235
PF20411	DUF6697	Domain of unknown function (DUF6697)	DPCfam:MC122485
PF20412	RALGAPB_N	RALGAPB N-terminal domain	DPCfam:MC15439
PF20413	Kiaa1109_N	Kiaa1109 N-terminal region	DPCfam:MC157111
PF20414	DUF6698	Family of unknown function (DUF6698)	DPCfam:MC60251
PF20415	DUF6699	Family of unknown function (DUF6699)	DPCfam:MC234314
PF20416	DUF6700	Domain of unknown function (DUF6700)	DPCfam:MC430631

PF20419	DUF6701	Family of unknown function (DUF6701)	DPCfam:MC93677
PF20420	DUF6702	Domain of unknown function (DUF6702)	DPCfam:MC91969
PF20515	2OG-FeII_Oxy_6	Tet-like 2OG-Fe(II) oxygenase superfamily	DPCfam:MC157368
PF20516	PDDEXK_12	PD-(D/E)XK nuclease superfamily	DPCfam:MC157427
