# Supplementary material for: DPCfam: Unsupervised protein family classification by Density Peak Clustering of large sequence datasets
Source: PLoS Comput Biol. 2022 Oct 19;18(10):e1010610. doi: 10.1371/journal.pcbi.1010610 (PMC9621593; doi:10.1371/journal.pcbi.1010610)
Supplement: S3 Table — (PDF) [file pcbi.1010610.s003.pdf]

ID	MC	seed size	Added to Pfam	AF representative	AF repr. start end pos	E-value	Coverage of MC's pHMM
TOP1	MC411630	2629	PF20151	//	//	//	//
TOP2	MC304269	2446	PF20152	//	//	//	//
TOP3	MC186369	1548	PF20150	AF-U7Q374-F1	13 - 181	3.6e-21	0.98
TOP4	MC385670	1210	PF20154	AF-Q39T32-F1	24 - 188	3.9e-39	1
TOP5	MC487727	1137	PF20153	AF-A0A0D2H4U1-F1	79 - 166	0.00043	0.41
TOP6	MC116910	1105	PF20155	AF-Q5F977-F1	97 - 319	1.2e-55	0.94
TOP7	MC297638	834	PF20148	AF-A0A077ZMA1-F1	44 - 130	1.2e-20	0.90
TOP8	MC202620	754	PF20147	AF-A0A2H5RJD4-F1	3 - 106	2.7e-27	0.98
TOP9	MC405686	694	PF20149	//	//	//	//
TOP10	MC15137	688	PF20146	AF-Q8IR42-F1	153 - 299	3.7e-32	0.95
TOP11	MC448731	683	PF20209	AF-A0A2R8RYX2-F1	594 - 733	5e-29	0.97
TOP12	MC163262	676	no	AF-K0EP06-F1	9 - 80	3.5e-28	0.97
TOP13	MC242539	670	no	AF-Q01960-F1	1 - 57	8.7e-20	0.92
TOP14	MC318107	667	no	AF-A0A1C1CS63-F1	7 - 248	1e-40	0.97
TOP15	MC24940	666	no	//	//	//	//
TOP16	MC234314	662	PF20415	AF-A0A1C1CTU8-F1	70 - 138	7e-06	0.58
TOP17	MC114843	657	no	AF-Q5HTX9-F1	156 - 497	4.5e-06	0.75
TOP18	MC199907	647	no	//	//	//	//
TOP19	MC430077	646	no	//	//	//	//
TOP20	MC392869	641	no	AF-P21296-F1	307 - 435	4.8e-17	0.99
TOP21	MC338779	624	no	AF-Q32CH7-F1	45 - 72	0.00023	0.47
TOP22	MC126457	620	PF20237	AF-A0A0D2GF18-F1	39 - 318	4.9e-63	0.98
TOP23	MC372031	611	no	//	//	//	//
TOP24	MC233206	599	no	//	//	//	//
TOP25	MC453907	598	PF20241	AF-Q109I2-F1	92 - 344	3.3e-88	0.99
