# Supplementary figures and images for: DPCfam: Unsupervised protein family classification by Density Peak Clustering of large sequence datasets
Source: PLoS Comput Biol. 2022 Oct 19;18(10):e1010610. doi: 10.1371/journal.pcbi.1010610 (PMC9621593; doi:10.1371/journal.pcbi.1010610)

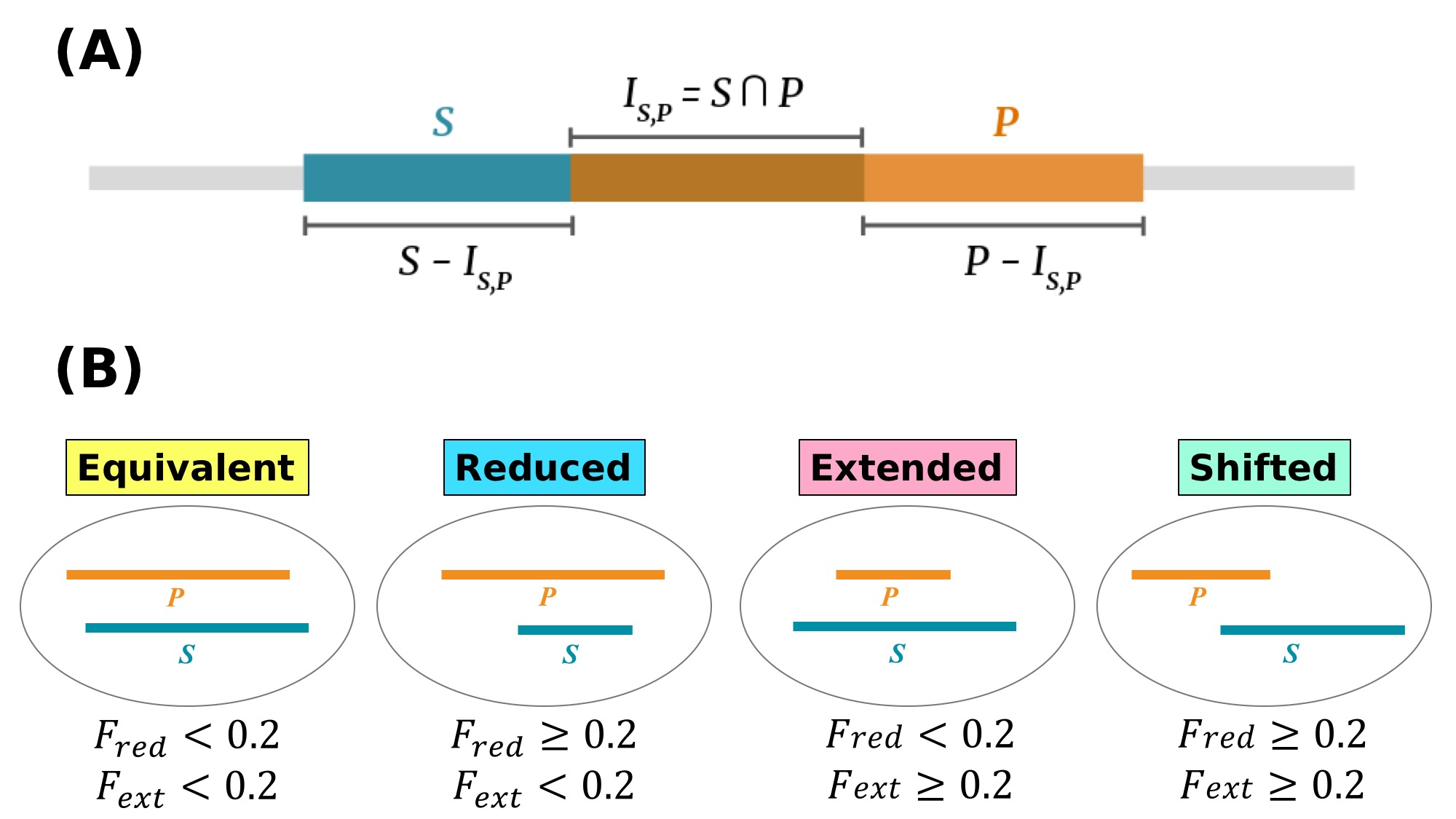

Supplement: S1 Fig — See Methods in main text. (TIF) [file pcbi.1010610.s004.tif]

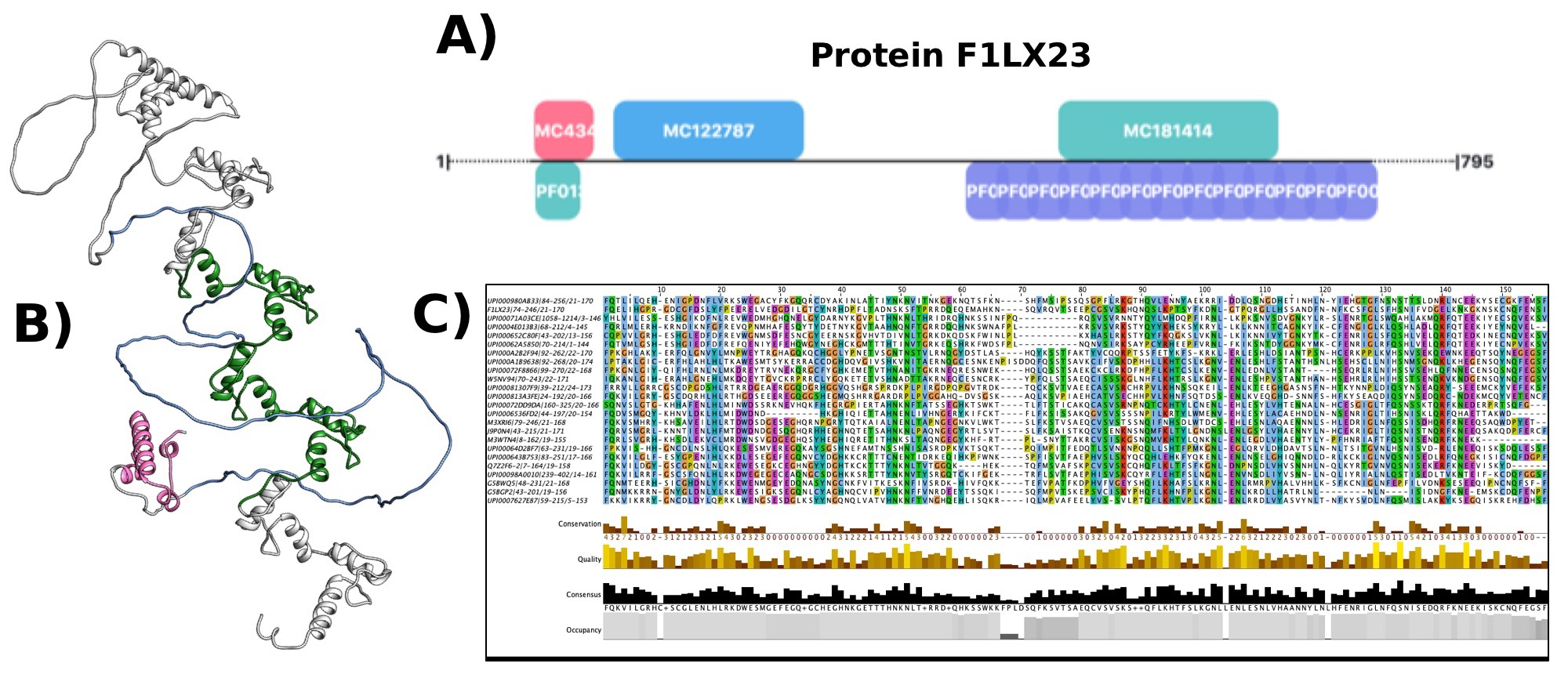

Supplement: S2 Fig — MC122787 has 41% of amino-acids in a disordered region and it is classified as an unknown MC, that is, none of its seed sequences carries a Pfam annotation. A) MC and Pfam family architecture of DNA-binding protein F1LX23, a representative of seed sequence of MC12287. The thin black like represents the full length protein sequence. Boxes above the sequence represent DPCfam MC annotations: MC434132 from aa 22 to aa 55 (pink), MC122787 from aa 74 to 246 (blue), MC181414 from aa 479 to 678 (green); boxes below the sequence represent instead Pfam family annotations: PF01352 (KRAB) from aa 3 to 43 (green), 13 repeats of PF00096 (zf-C2H2) from aa 395 to 753 (purple) (the image is generated by the DPCfam website, https://dpcfam.areasciencepark.it/). B) Three-dimensional structure of protein F1LX23 as predicted by AlphaFold2 (Cramer, Patrick. “AlphaFold2 and the future of structural biology.” Nature Structural & Molecular Biology 28(9) (2021): 704–705.). Colors indicate where DPCfam annotations map onto the protein structure (color scheme is the same as the one used in A) for MCs). It can be seen that MC12287 (blue) is predicted to be in an unstructured region. C) MC122787’s multiple sequence alignment with conservation, quality, consensus and occupancy histograms. Note that while only a few representative sequences are shown, conservation and consensus are calculated on the full set of aligned sequences. Figure drawn with Jalview (Waterhouse AM, Procter JB, Martin DMA, Clamp M, Barton GJ “Jalview Version 2-a multiple sequence alignment editor and analysis workbench”. Bioinformatics 25 (2009): 1189–1191.). (TIF) [file pcbi.1010610.s005.tif]

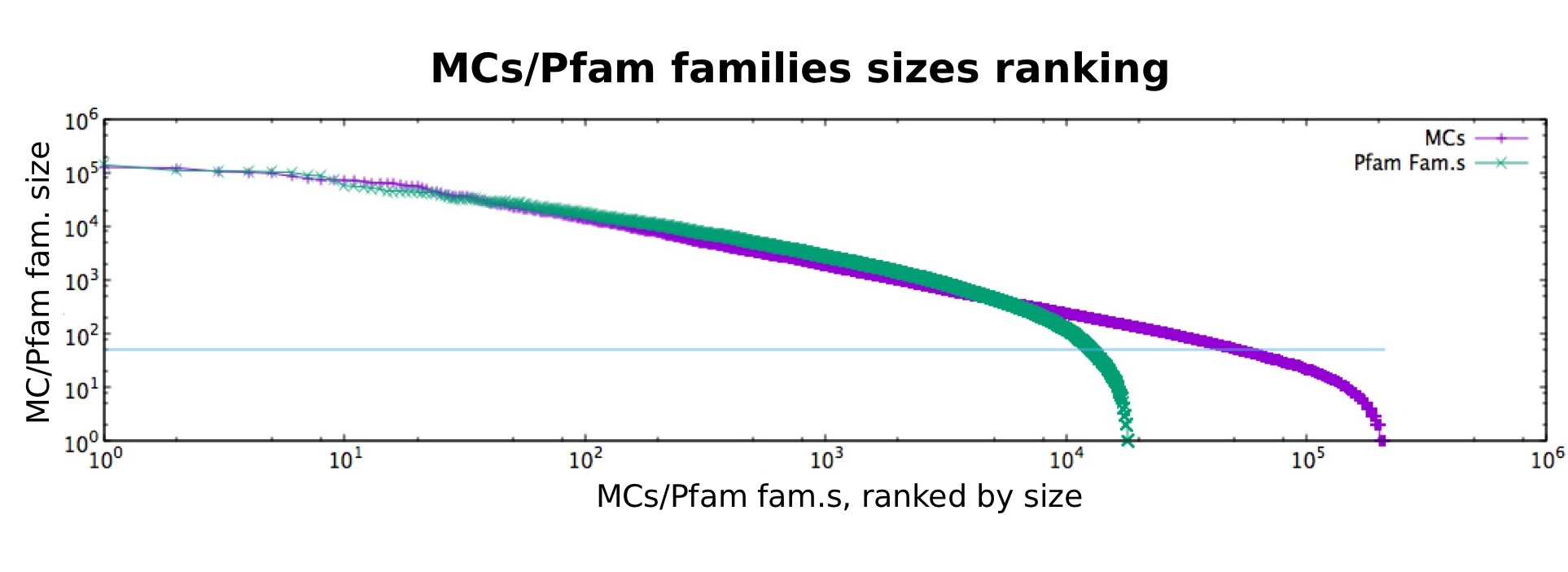

Supplement: S3 Fig — MC size is calculated as the number of seed sequences. Pfam families (v. 32) are all those in Uniref50 ∩ UniprotKB (18,189 total) and family size is the ‘full’ size, that is, not only considering Pfam seed sequences. The blue horizontal line corresponds to a size of 50; MCs smaller than 50 have not been considered in the analysis presented in this work. (TIF) [file pcbi.1010610.s006.tif]

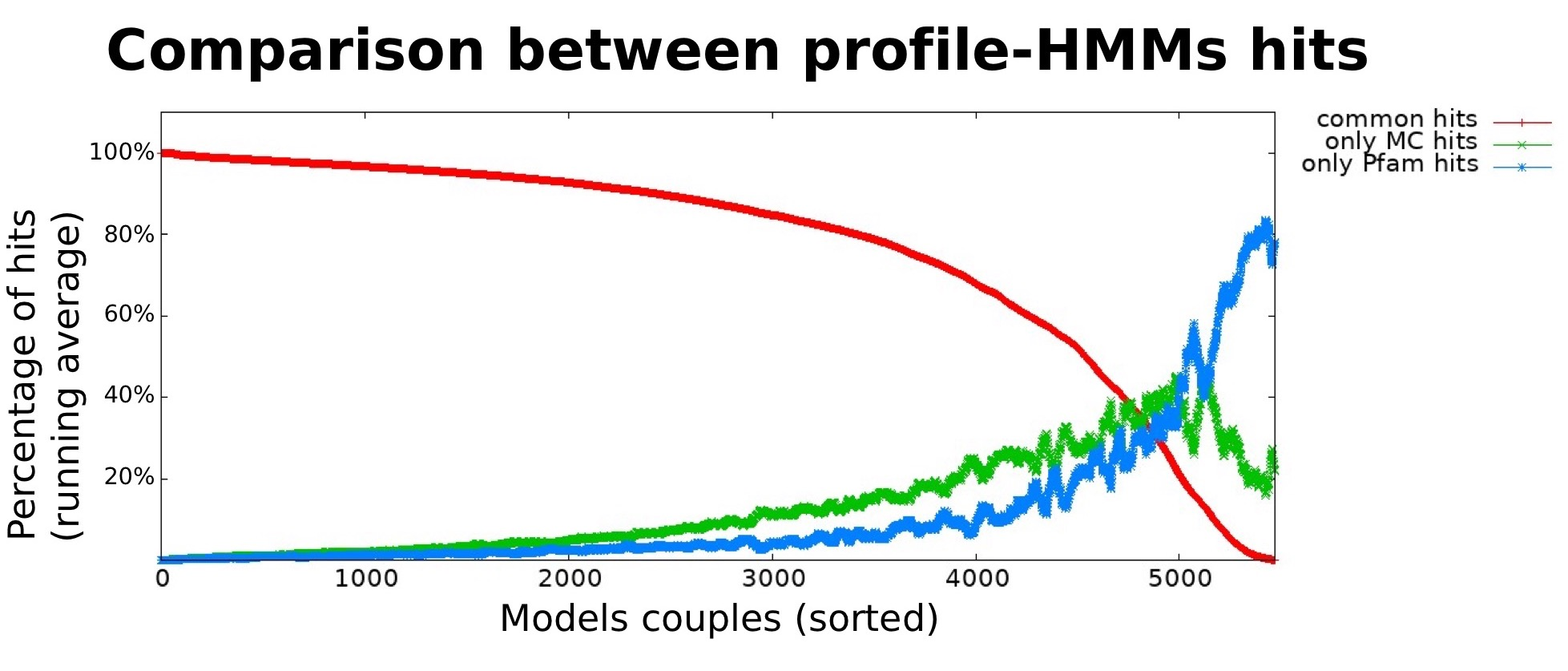

Supplement: S4 Fig — The red curve shows the fraction of common protein hits between the profile-HMM models in each pair (running average, window of size 50), note that in this case we compare protein IDs with no specific requirement for overlap between hits to the same ID; the other curves represent the fraction of protein hits that are found only by the DPCfam (green) or the Pfam (blue) models, respectively (running averages as above). For each MC-Pfam family pair, fractions are computed with respect to the union of all protein hits found by the MC and the Pfam model. Pairs along the x-axis are sorted by decreasing fraction of common hits. About 60% of profile-HMMs from equivalent MC-family pairs share more than 80% of their UniRef50 hits and about 80% of them share more than half of their hits. For around 9.7% of equivalent profile-HMMs, the fraction of shared hits is instead less than 25%. On average, these latter profile-HMMs appear to represent large, diverse families, for which DPCfam and Pfam tend to capture different member regions. In fact, the average size of Pfam families sharing less than 25% of hits with their associated equivalent MC is 2,562, this is to be compared to an overall average size of 800. (TIF) [file pcbi.1010610.s007.tif]

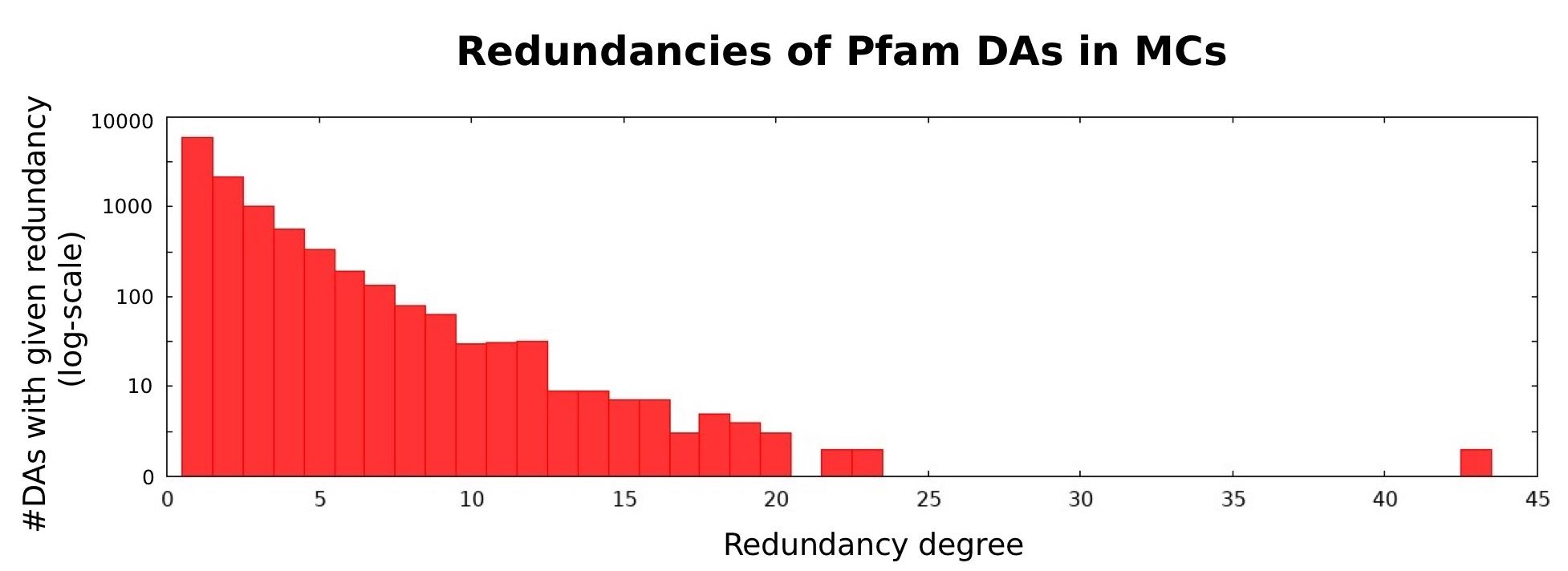

Supplement: S5 Fig — (TIF) [file pcbi.1010610.s008.tif]

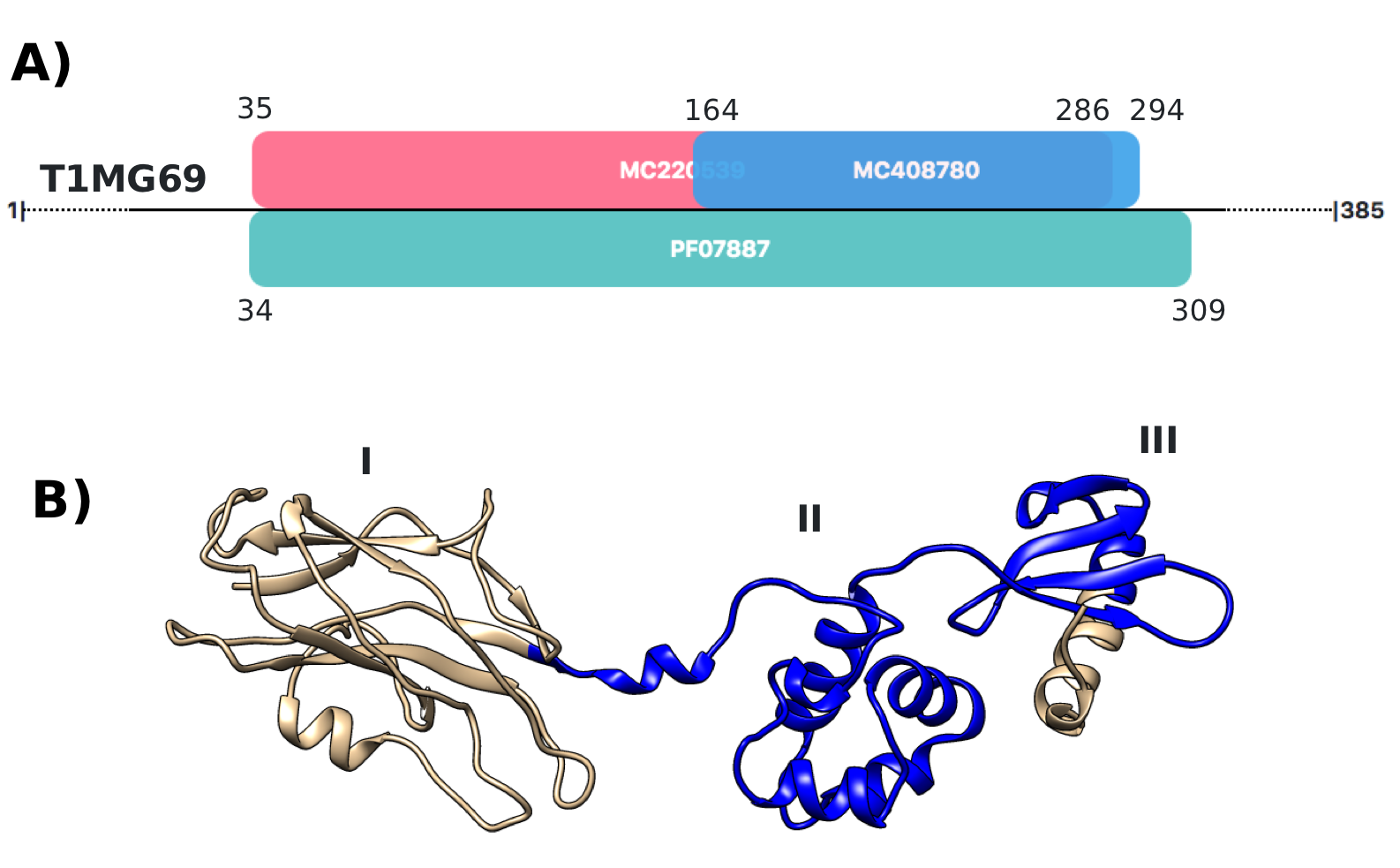

Supplement: S6 Fig — A: location of a SSR from MC220539 (pink), a SSRs from MC408780 (blue) and Pfam family PF07887 (pale-blue), on protein T1MG69. MC220539 and MC408780 both feature PF07887 (Calmodulin binding protein-like) as DA, the former with “equivalent” boundaries, the latter with “reduced” boundaries. B: structural prediction of PF07887 by trRosetta (from the Pfam website), with highlighted in blue the position of the “reduced” MC (MC408780), as found by running the MC profile-HMM with HHpred (standard parameters) against the T1MG69 sequence. As noted in the Pfam blog-post “Folding the protein universe” (31 march 2021, see https://xfam.wordpress.com/2021/03/03/folding-the-protein-universe/), structural prediction of the Calmodulin binding protein-like family shows three separate structural domains (I, II and III approximately). Indeed, in Pfam version 35 this family has been split in three different families. It can be seen that two of these domains have close correspondence to the “reduced” MC (MC408780). (TIF) [file pcbi.1010610.s009.tif]

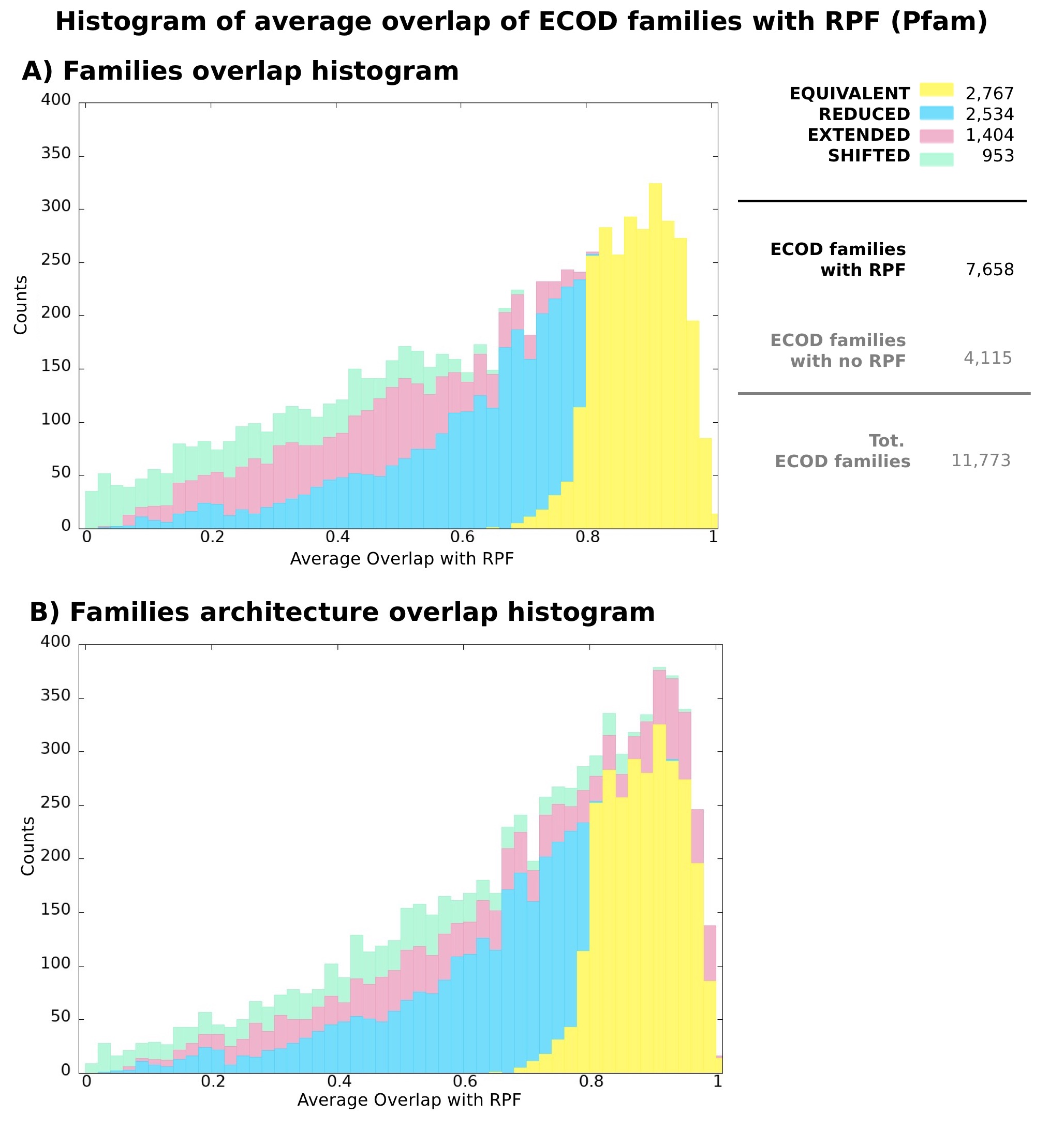

Supplement: S7 Fig — Columns are colored according to the RPF boundary classification (equivalent, reduced, extended and shifted). A: Overlap between individual ECOD families and their RPF. B: Overlap between individual ECOD families or architectures and RPFs. Given an ECOD family and its RPF (same pairs as in A), we search for a better overlap of the RPF with any multi-family architecture featuring the original ECOD family and up to two additional ECOD families. The reported average overlap value is thus the best between the overlap with the original family and any other such ECOD architecture. Note that the ECOD architecture labels (equivalent/reduced/extended/shifted) are still assigned according to the RPF overlap to the original ECOD family so as to show to which extent the overlap in each ECOD family category increases with respect to A). (TIF) [file pcbi.1010610.s010.tif]

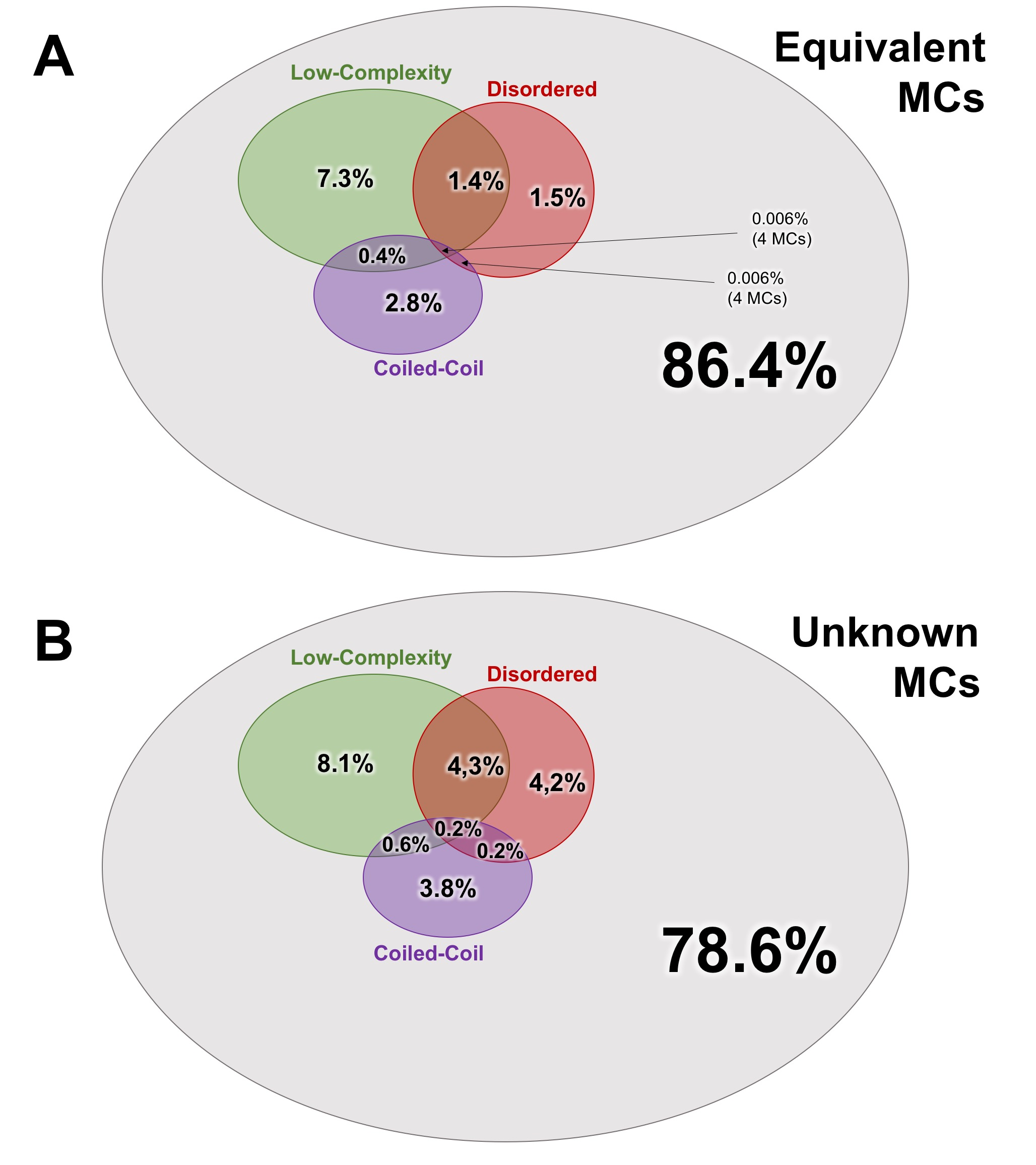

Supplement: S8 Fig — Venn diagrams showing the percentage of Low Complexity MCs (green), Disordered MCs (red) and Coiled-Coil MCs (purple), respectively (see Methods for definitions) in A) Equivalent MCs (equivalent to a Pfam family, Methods). Panel B: MCs unknown to Pfam. 86.4% and 78.6% is the number of MCs featuring none of the above regions among equivalent and unknown MCs, respectively. (TIF) [file pcbi.1010610.s011.tif]

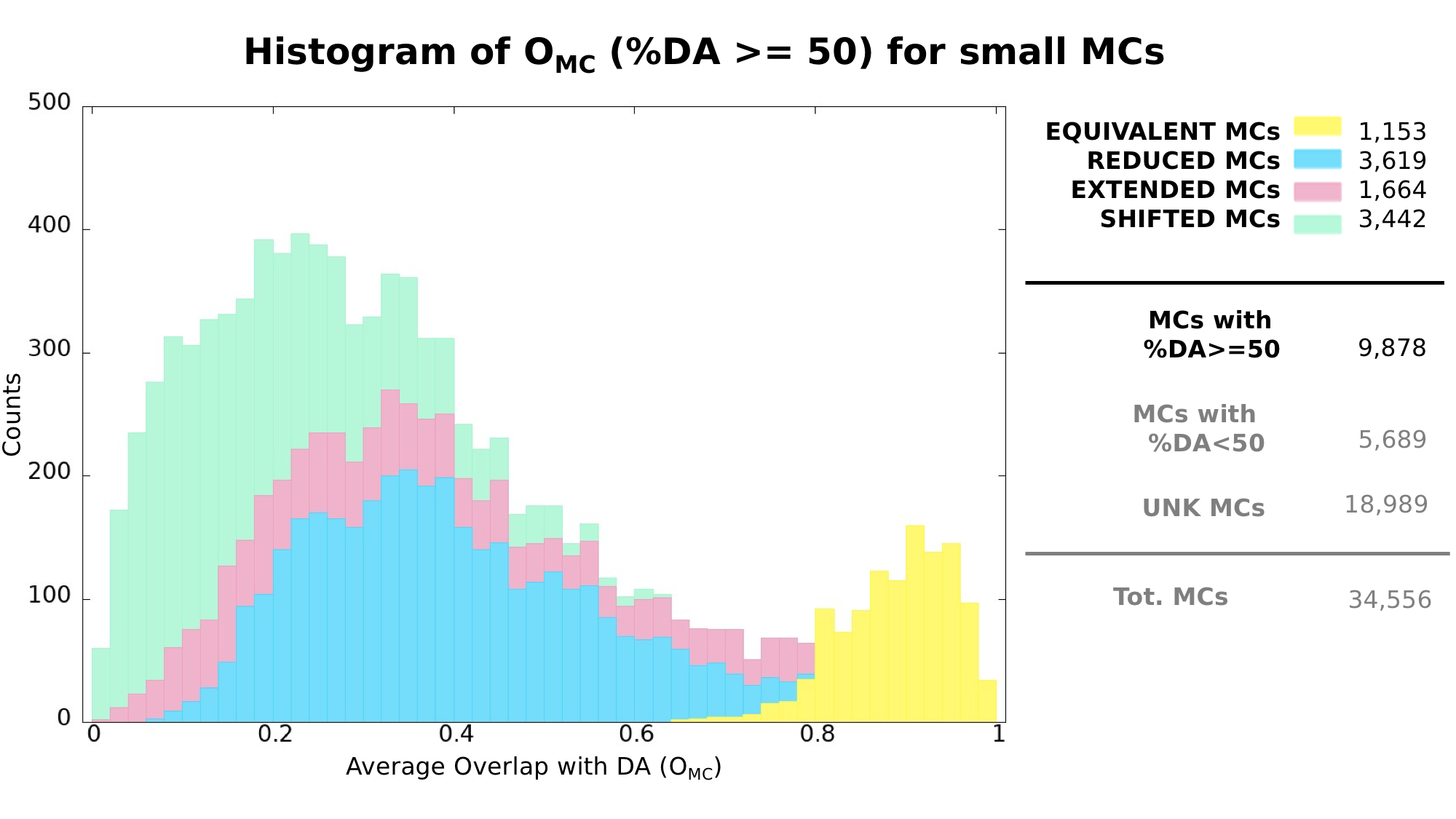

Supplement: S9 Fig — Colors reflect the contribution of each MC category to each bin (equivalent, reduced, extended and shifted, see also S1B Fig). The legend on the right side of the histogram reports total counts of MCs in each category, total count of MCs with %DA< 50 and total count of unknown MCs. (TIF) [file pcbi.1010610.s012.tif]

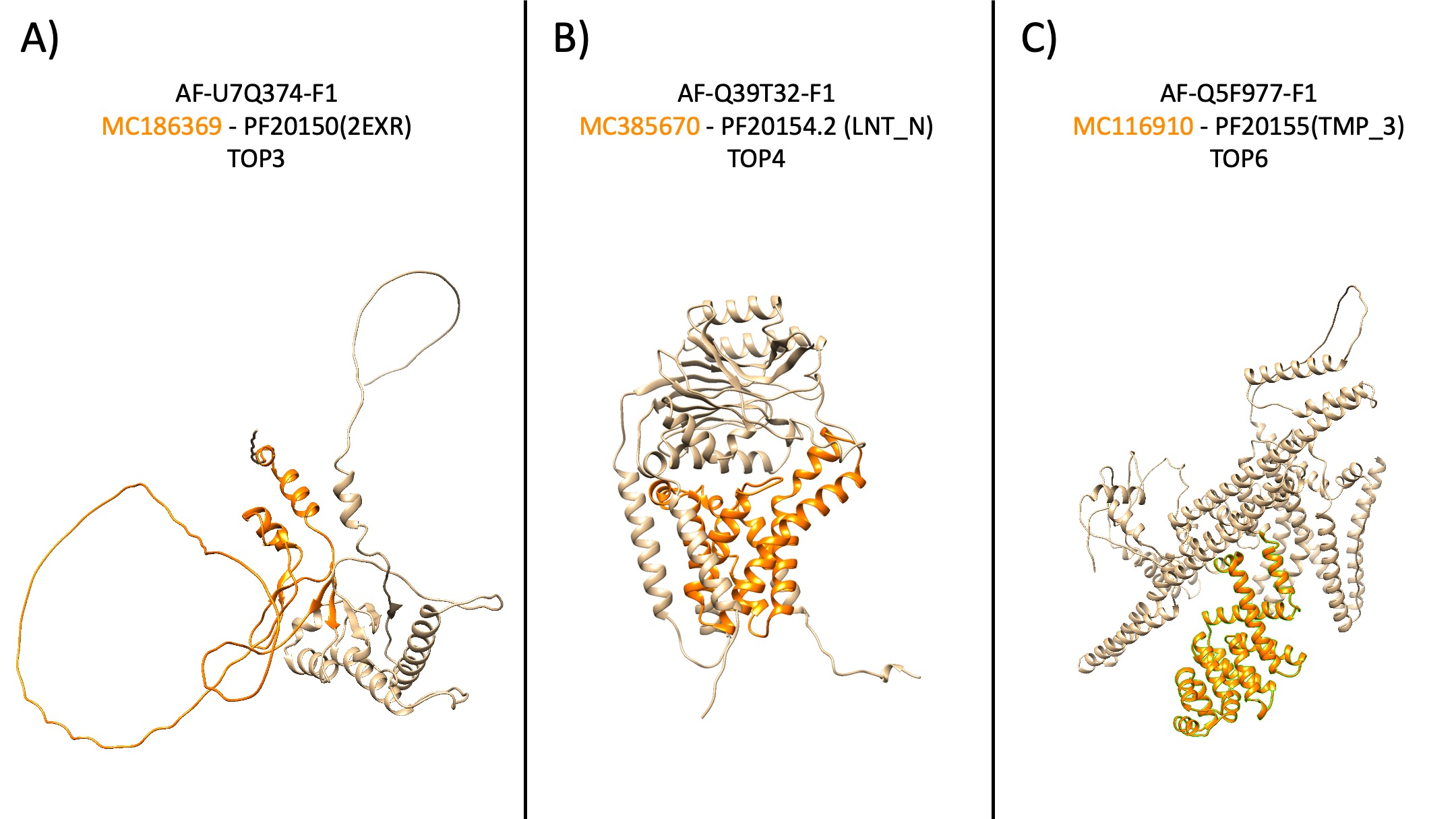

Supplement: S10 Fig — The part of the sequence of the AlphaFold protein that aligns to the MC profile-HMM is shown in orange. Above each example we report the ID of the AlphaFold structure, the MC number, the Pfam family that was build from the MC (if any) and the position of the MC in the list of largest unknown MCs (S3 Table). Molecular graphics and analyses performed with UCSF Chimera (Pettersen EF, Goddard TD, Huang CC, Couch GS, Greenblatt DM, Meng EC, Ferrin TE. “UCSF Chimera–a visualization system for exploratory research and analysis.” J Comput Chem. 25 (2004):1605–12). (TIF) [file pcbi.1010610.s013.tif]

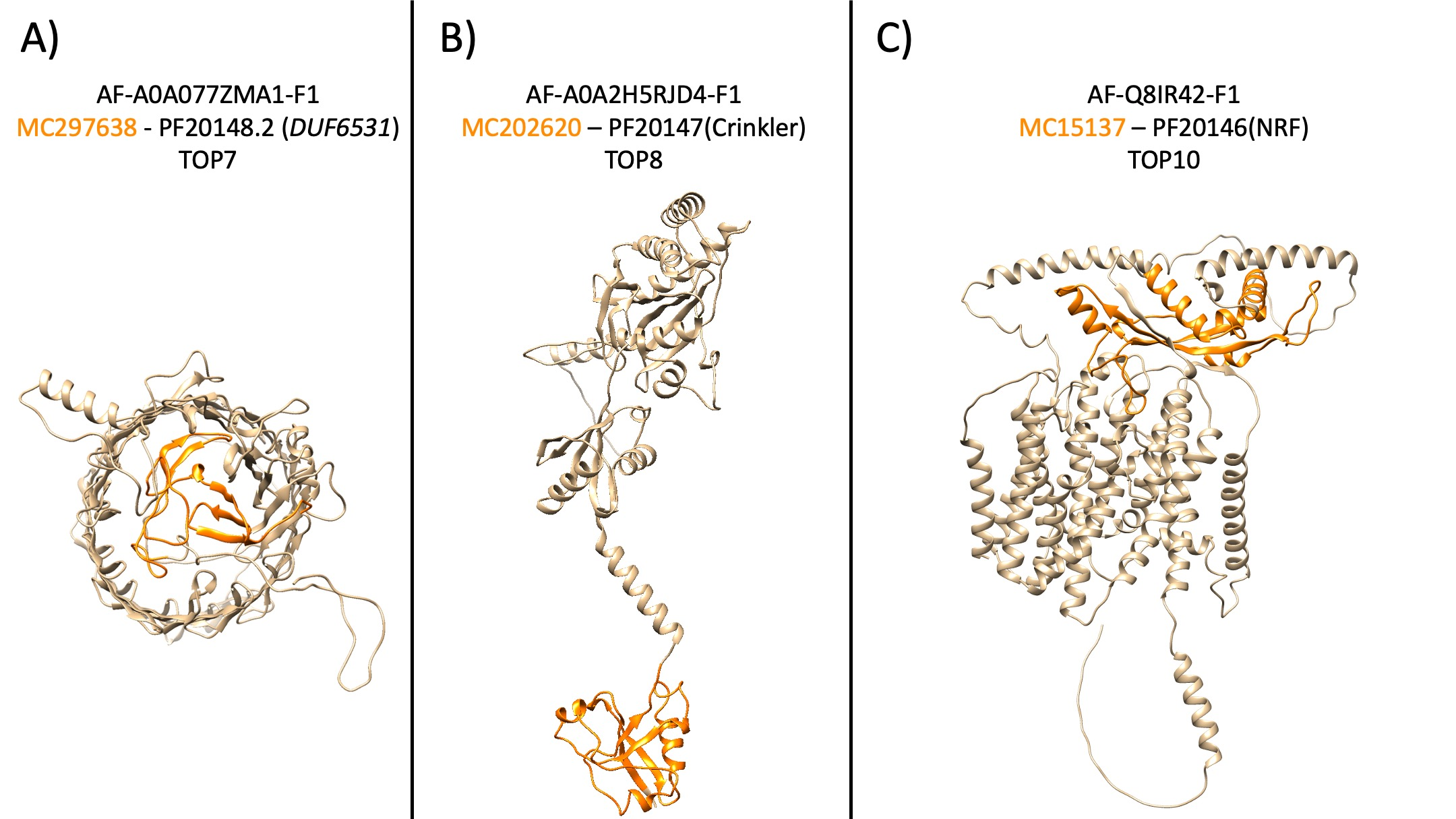

Supplement: S11 Fig — The part of the sequence of the AlphaFold protein that aligns to the MC profile-HMM is shown in orange. Above each example we report the ID of the AlphaFold structure, the MC number, the Pfam family that was build from the MC (if any) and the position of the MC in the list of largest unknown MCs (S3 Table). Molecular graphics and analyses performed with UCSF Chimera (Pettersen EF, Goddard TD, Huang CC, Couch GS, Greenblatt DM, Meng EC, Ferrin TE. “UCSF Chimera–a visualization system for exploratory research and analysis.” J Comput Chem. 25 (2004):1605–12). (TIF) [file pcbi.1010610.s014.tif]

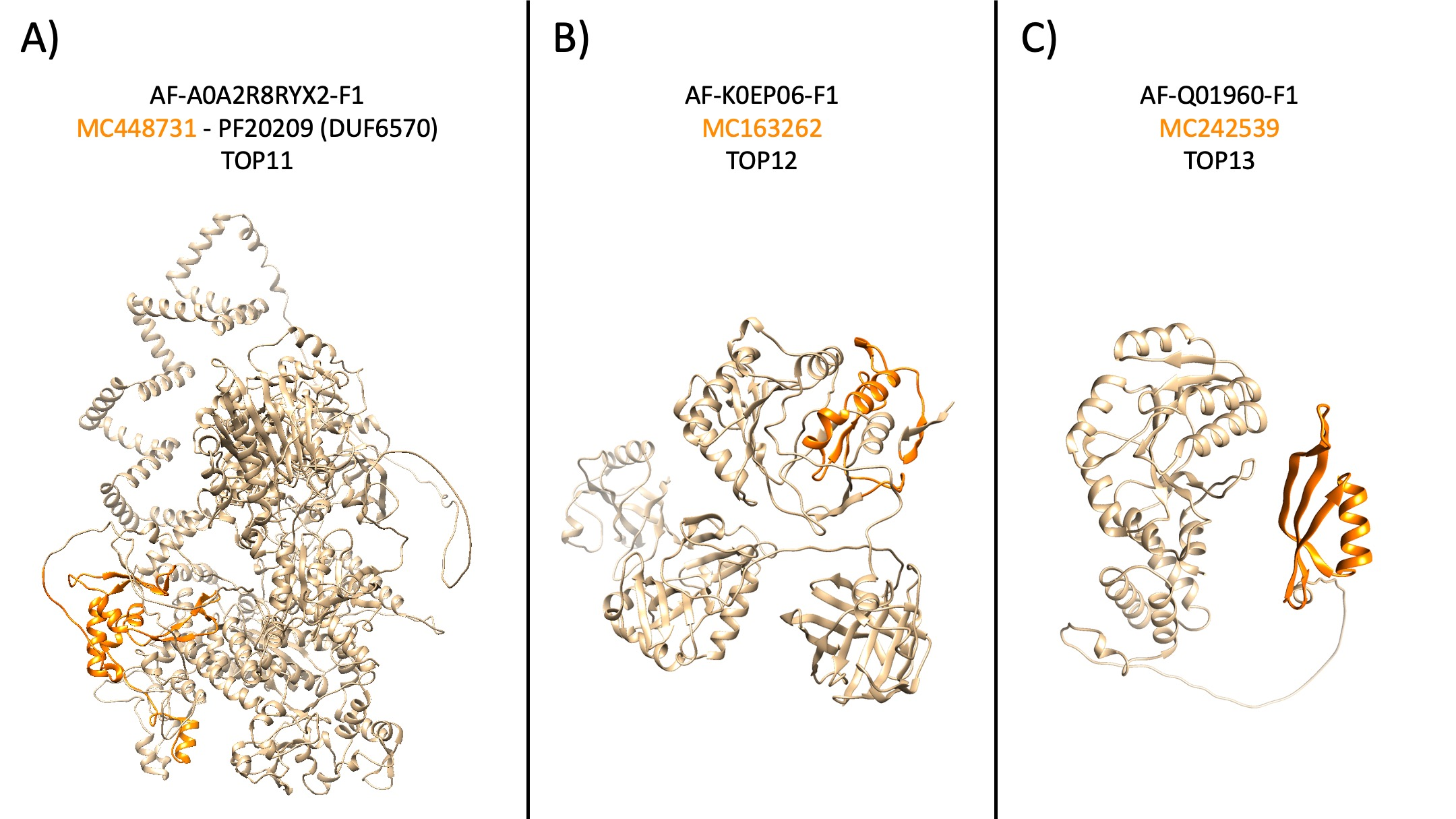

Supplement: S12 Fig — The part of the sequence of the AlphaFold protein that aligns to the MC profile-HMM is shown in orange. Above each example we report the ID of the AlphaFold structure, the MC number, the Pfam family that was build from the MC (if any) and the position of the MC in the list of largest unknown MCs (S3 Table). Molecular graphics and analyses performed with UCSF Chimera (Pettersen EF, Goddard TD, Huang CC, Couch GS, Greenblatt DM, Meng EC, Ferrin TE. “UCSF Chimera–a visualization system for exploratory research and analysis.” J Comput Chem. 25 (2004):1605–12). (TIF) [file pcbi.1010610.s015.tif]

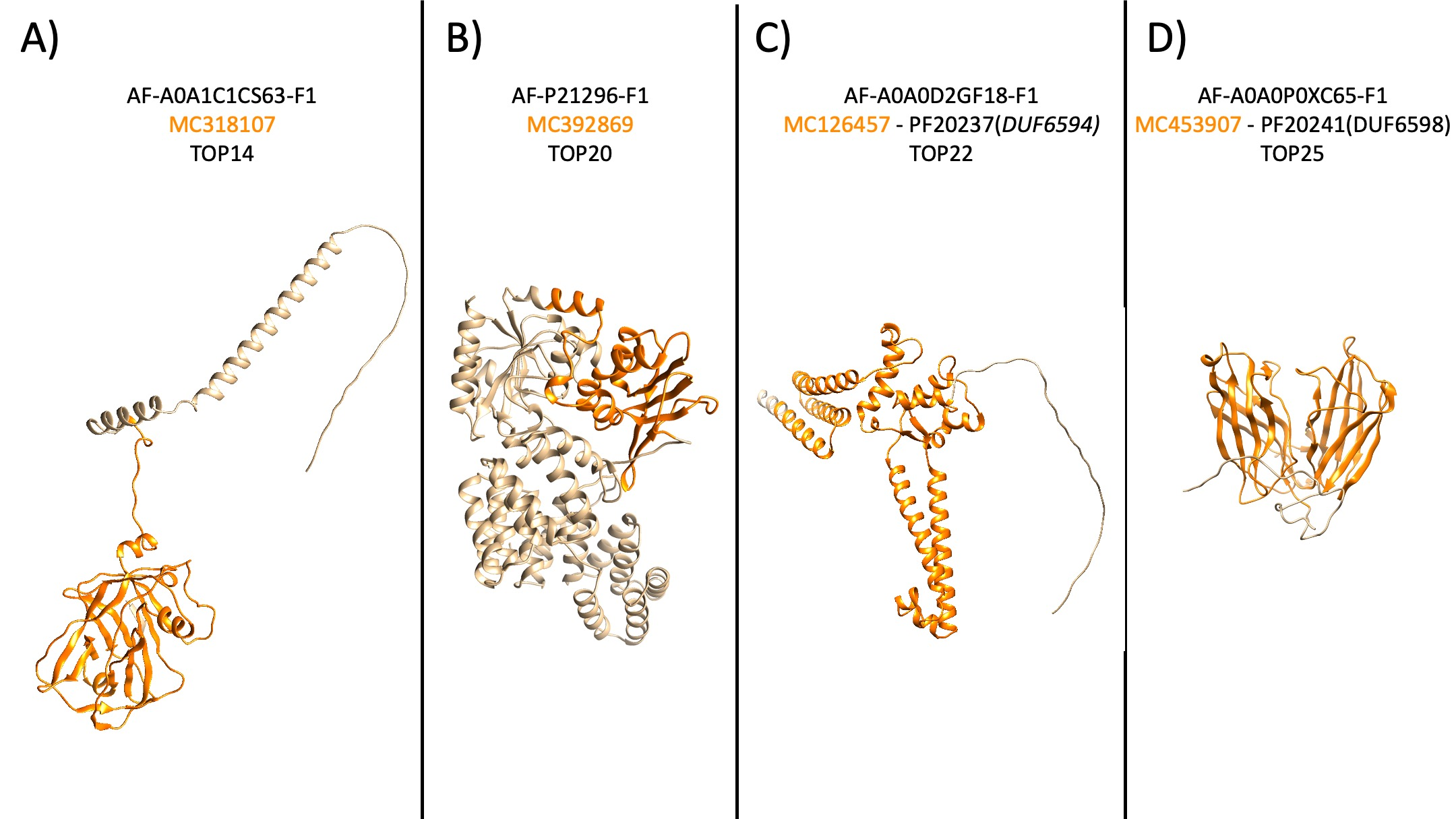

Supplement: S13 Fig — The part of the sequence of the AlphaFold protein that aligns to the MC profile-HMM is shown in orange. Above each example we report the ID of the AlphaFold structure, the MC number, the Pfam family that was build from the MC (if any) and the position of the MC in the list of largest unknown MCs (S3 Table). Molecular graphics and analyses performed with UCSF Chimera (Pettersen EF, Goddard TD, Huang CC, Couch GS, Greenblatt DM, Meng EC, Ferrin TE. “UCSF Chimera–a visualization system for exploratory research and analysis.” J Comput Chem. 25 (2004):1605–12). (TIF) [file pcbi.1010610.s016.tif]

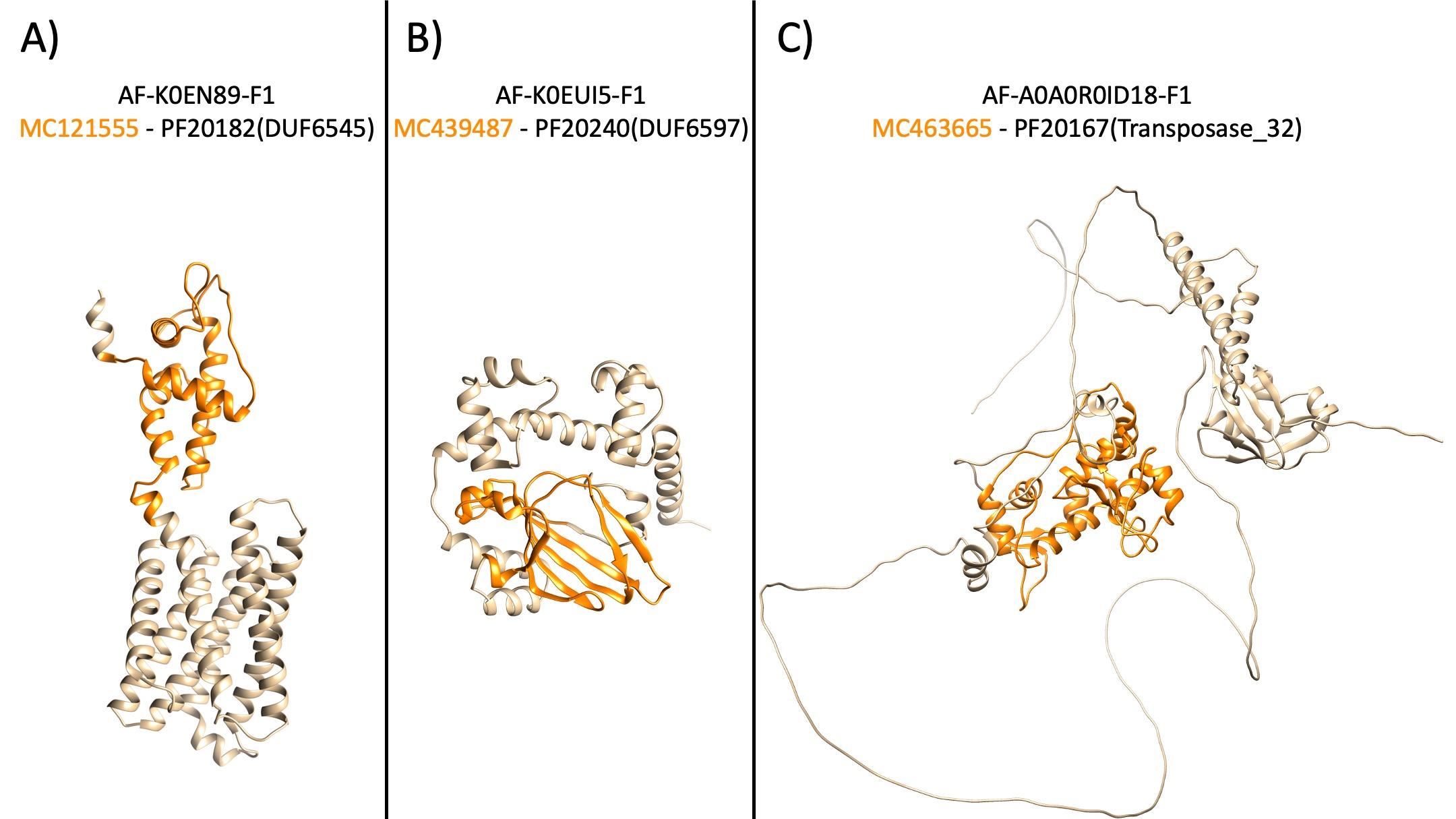

Supplement: S14 Fig — The part of the sequence of the AlphaFold protein that aligns to the MC profile-HMM is shown in orange. Above each example we report the ID of the AlphaFold structure, the MC number, the Pfam family that was build from the MC (if any). Molecular graphics and analyses performed with UCSF Chimera (Pettersen EF, Goddard TD, Huang CC, Couch GS, Greenblatt DM, Meng EC, Ferrin TE. “UCSF Chimera–a visualization system for exploratory research and analysis.” J Comput Chem. 25 (2004):1605–12). (TIF) [file pcbi.1010610.s017.tif]
